# Integrated Transcriptomics Reveals Evolutionary Trajectories and Cell Density‐Dependent Mechanisms in Aldosterone‐Producing Adenomas

**DOI:** 10.1002/advs.202505410

**Published:** 2026-01-21

**Authors:** Zhuolun Sun, Changying Jing, Martina Tetti, Siyuan Gong, Jia Wei, Yingxian Pang, Martin Reincke, Tracy Ann Williams

**Affiliations:** ^1^ Medizinische Klinik und Poliklinik IV Klinikum der Universität München Ludwig‐Maximilians‐Universität München 80336 Munich Germany; ^2^ Institute of Diabetes and Regeneration Research Helmholtz Munich Neuherberg, Germany, German Center for Diabetes Research (DZD) Neuherberg, Germany Munich Medical Research School Ludwig‐Maximilians‐Universität 80539 Munich Germany

**Keywords:** Aldosterone‐producing adenomas, evolutionary trajectory, single‐cell/nucleus transcriptomics, spatial transcriptomics, tumor microenvironment

## Abstract

Aldosterone‐producing adenomas (APAs) and micronodules (APMs) are histopathological lesions that can cause a prevalent form of secondary hypertension called primary aldosteronism. Using integrated single‐cell/nucleus and spatial transcriptomics, it demonstrates substantial heterogeneity between APMs and APAs, alongside an immunosuppressive tumor microenvironment in APAs. Pseudotime analyses reveal two distinct evolutionary trajectories in APAs: a direct progression from zona glomerulosa cells to APAs, observed specifically in *KCNJ5*‐mutated tumors, and a stepwise path from zona glomerulosa cells to APMs and then to APAs, present in both *KCNJ5*‐mutated APAs and APAs without *KCNJ5* mutations. Within *KCNJ5*‐mutated APAs, we identified two distinct cellular states with different differentiation potentials: a progenitor‐like state (high differentiation potential) and a mature state (low differentiation potential). This uncovered a spectrum of molecular and functional shifts during APA progression involving pathways of oxidative stress (including mechanisms leading to cell death by ferroptosis) and focal adhesion (influenced by cell density). In vitro studies in human adrenal cells demonstrate that *TAZ* activation regulates ferroptosis sensitivity in response to cell density changes. The findings enhance understanding of the transcriptional diversity in APMs and APAs and elucidate complex molecular alterations underlying APA pathogenesis.

## Graphical abstract

Aldosterone‐producing adenomas (APAs) develop via two distinct paths: directly from adrenal zona glomerulosa (zG) cells, or stepwise from zG cells through aldosterone‐producing micronodules (APMs) before progressing to APAs. Advanced single‐cell and spatial analyses identified distinct cell states linked to oxidative stress and cell–cell interactions, while TAZ protein regulates sensitivity to stress‐induced cell death, revealing key mechanisms in APA pathogenesis.

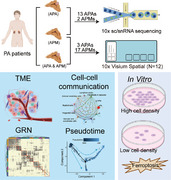

## 1 Introduction

Aldosterone, produced by zona glomerulosa (zG) cells in the adrenal cortex, is crucial for electrolyte balance and blood volume homeostasis.^[^ ^1^ ^]^ In primary aldosteronism (PA), a common cause of secondary hypertension, aldosterone is secreted autonomously, leading to excessive production and clinical implications such as resistant hypertension and increased cardiovascular risk.^[^ ^2^, ^3^ ^]^ PA affects ≈6% of the general hypertensive population, rising to 10–20% in specialized hypertension centers.^[^ ^4^, ^5^, ^6^ ^]^

PA is typically categorized into unilateral aldosterone‐producing adenomas (APA) or bilateral adrenal hyperplasia.^[^ ^2^, ^7^ ^]^ Some cases of unilateral PA lack a discernible APA, instead exhibiting subcapsular aldosterone producing micronodules (APMs).^[^ ^8^, ^9^ ^]^ APAs and APMs can be visualized using CYP11B2 (aldosterone synthase) immunohistochemistry and there is some, albeit limited, evidence from molecular histopathology and spatial transcriptomics that supports the hypothesis that, in certain cases, an APM may progress to an APA.^[^ ^10^, ^11^ ^]^ Somatic driver mutations have been identified in nearly all APAs, affecting genes encoding ion channels (*KCNJ5, CACNA1D, CACNA1H, CLCN2*),^[^ ^12^, ^13^, ^14^, ^15^, ^16^ ^]^ transporters (*ATP1A1, ATP2B3, SLC30A1*),^[^ ^14^, ^17^, ^18^ ^]^ and other proteins (*CTNNB1, CADM1, GNAS*).^[^ ^19^, ^20^, ^21^, ^22^ ^]^ *KCNJ5* mutations occur more frequently than others.^[^ ^3^, ^20^ ^]^ These PA‐driver mutations generally activate the calcium signaling pathway, enhancing *CYP11B2* gene expression resulting in increased aldosterone production. APMs are found in both healthy and PA adrenal glands.^[^ ^23^, ^24^ ^]^ They frequently carry mutations in *CACNA1D, ATP1A1*, or *ATP2B3*, but rarely in *KCNJ5*.^[^ ^2^, ^8^, ^10^, ^25^, ^26^ ^]^

Although single‐cell and single‐nucleus RNA sequencing (sc/snRNA‐seq) have revealed cellular heterogeneity and progression trajectories in adrenal tissues,^[^ ^27^, ^28^, ^29^, ^30^ ^]^ these studies have been limited to a single histopathological lesion by focusing either on APMs or APAs. Spatial transcriptomics has further enabled simultaneous acquisition of spatial organization and transcriptome data in APAs.^[^ ^31^ ^]^ However, studies exploring the global cellular landscape and interaction network differences between APMs and APAs remain scarce. We hypothesize that APAs develop via multiple pathways involving alterations in cellular composition, tumor microenvironment remodeling, gene regulatory network restructuring, and metabolic adaptations. To test this, we employed single‐cell, single‐nucleus, and spatial transcriptomics technologies to construct a high‐resolution map of the cellular and molecular landscapes in APA progression.

Our integrated approach revealed distinct phenotypic profiles and diverse cell type compositions between APMs and APAs. Trajectory analysis identified two potential developmental pathways: a direct progression from zG cells to APAs, observed specifically in *KCNJ5*‐mutated tumors, and a stepwise path from zG cells to APMs and then to APAs, present in both APAs with and without *KCNJ5* mutations. APAs exhibited an altered tumor microenvironment associated with immunosuppression, potentially driven by promotion of M2 macrophage polarization. Reconstruction of gene regulatory networks elucidated how transcription factors orchestrate cellular state transitions during APA development. Furthermore, our analysis revealed that these cellular transitions are influenced by cell density, which modulates ferroptosis sensitivity in human adrenocortical cells. Additionally, we identified the Hippo pathway effector *TAZ* as a key regulator in this process. These findings elucidate the transcriptomic evolution during APA initiation and progression, uncovering potential molecular mechanisms underlying APA pathogenesis and offering new insights into adrenocortical tumorigenesis.

## 2 Results

### 2.1 Cell Type Composition and Diversity in APAs and APMs

**Figure 1:**
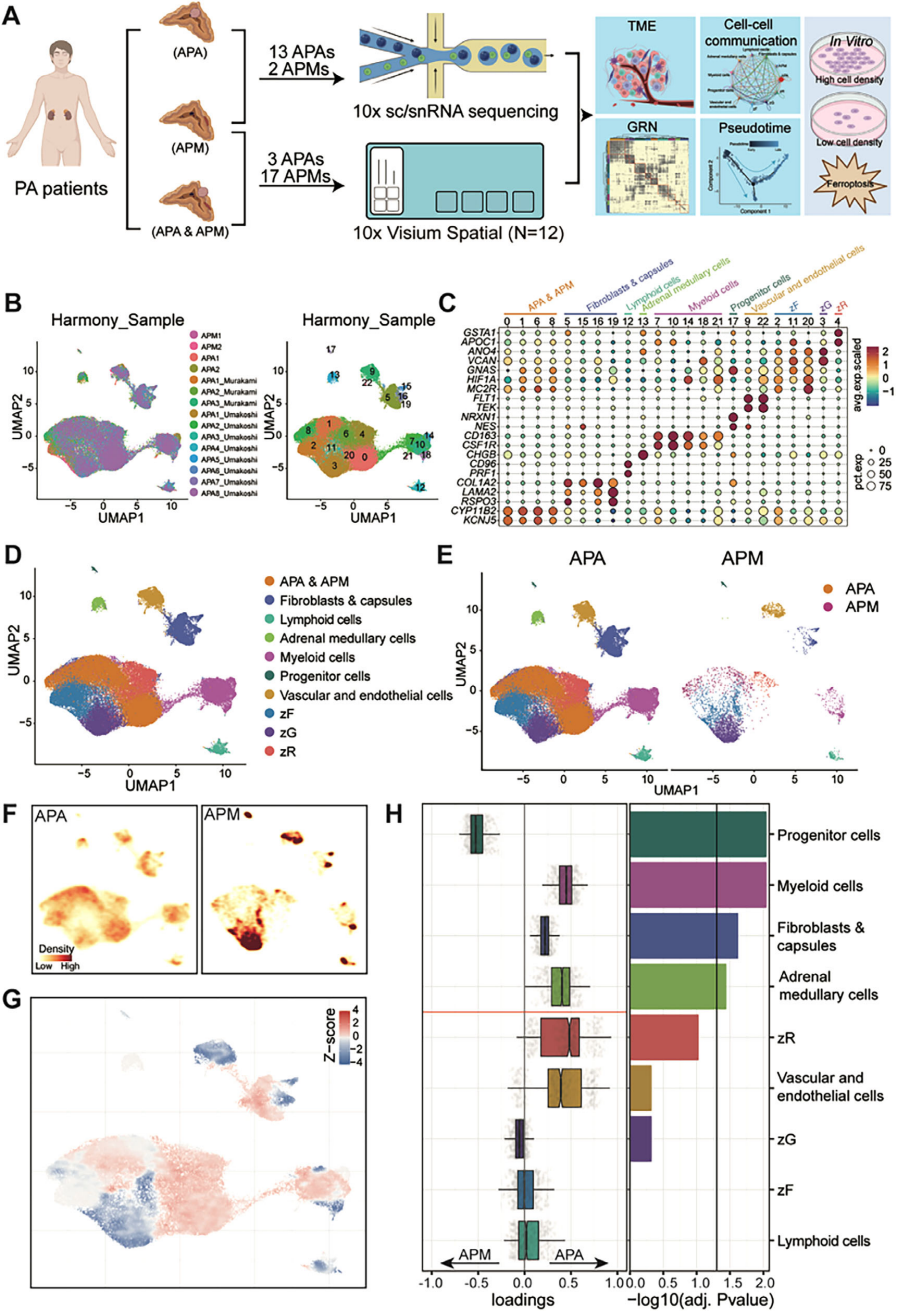
Global cellular landscape in human APA and APM tissues. A) Schematic overview of the experimental design and analytical workflow. B) Integrative analysis of 13 APA tissues and 2 APM samples, visualized using a common UMAP embedding for individual samples (left) and cell clusters (right). C) Bubble plot of representative cell‐type specific markers across all clusters. D) UMAP visualization of major cell types. E) Cell‐type distribution across APA (left) and APM (right) samples, visualized separately using UMAP. F) Changes in composition across all compartments and sample fractions are visualized as cell density on a unified embedding plot. G) Statistical assessment of cell density differences between APA and APM. A two‐sided Wilcoxon test was performed, with results visualized as Z scores. Red indicates increased cell abundance in APA, while blue indicates decreased cell abundance in APM. H) Changes in cell composition were assessed using Compositional Data Analysis. The x‐axis represents the separation coefficient for each cell type, where positive values indicate increased abundance in the APA and negative values signify decreased abundance. Boxplots and individual data points depict uncertainty derived from bootstrap resampling of samples and cells. Bar plots display the −log10 (adjusted P value) for the enrichment of major cell types. APA, aldosterone‐producing adenoma; APM, aldosterone‐producing micronodule; GRN, gene regulation network; PA, primary aldosteronism; sc/scRNA sequencing: single‐cell and single‐nucleus RNA sequencing; TME, tumor microenvironment; zG, zona glomerulosa; zF, zona fasciculata; zR, zona reticularis.

To generate an expression atlas of APA and APMs, we conducted snRNA‐seq on 2 APAs from male patients who had undergone adrenalectomy for unilateral PA (Table S1, Supporting Information). These APAs harbored different somatic mutations: one in *KCNJ5* and the other in *ATP1A1* (Tables S2 and S3, Supporting Information). To address the limited number of APA samples, we additionally incorporated two publicly available snRNA‐seq or scRNA‐seq data from 11 additional APA samples with a *KCNJ5* mutation (syn6023883, n = 8; GSE242404, n = 3).^[^ ^28^, ^30^ ^]^ For comparative analysis of cell‐type populations between APAs and APMs, we integrated all 13 APA samples with publicly available scRNA‐seq data from two APM samples (E‐MTAB‐11837) (**Figure** **1**A)^[^ ^27^ ^]^ of unknown genotype status (Table S4, Supporting Information).

Using an established Seurat‐based data preprocessing and clustering analysis pipeline, we identified a total of 61744 high‐quality transcriptomes, comprising 4438 cells from the APM samples and 57306 cells/nuclei from the APA samples. To assess the effectiveness of data integration, we evaluated five different methods for batch effect correction. Using the Uniform Manifold Approximation and Projection (UMAP) algorithm for visualization, we determined that of these methods, Harmony most effectively minimized batch effects across the 15 samples (Figure 1B; Figure S1A, Supporting Information).

Subsequently, unsupervised graph‐based clustering revealed 23 distinct clusters, encompassing a diverse array of cell types. These included APA & APM cells, adrenocortical cells (zG, zona fasciculata, and zona reticularis), adrenal medullary cells, progenitor cells, lymphoid cells, myeloid cells, vascular and endothelial cells, and fibroblasts & capsules (Figure 1B–D). Notably, APA and APM cells exhibited similar gene expression marker profiles, resulting in their initial co‐clustering. For a more refined classification of these closely related yet distinct cell populations, we reclassified them based on sample origin (Figure 1E).

Sample contributions to each cell type varied, but most cell types were derived from a relatively even distribution of samples (Figure S1B; TableS4, Supporting Information). To analyze differences in cell populations, we employed two complementary methods: cell density analysis on the joint UMAP embedding and direct comparison of cell proportions. Our global analysis revealed an enrichment of myeloid cells in APAs, along with increases in fibroblasts & capsule cells (Figure 1F,G). To address potential biases arising from proportional changes in one cell type affecting the representation of others, we implemented compositional data analysis to provide a more accurate estimation of relative changes in cell type composition across samples (Figure 1H). Furthermore, genotype‐based subgroup analysis revealed that the *ATP1A1*‐mutated APA harbored increased proportions of vascular and endothelial cells as well as fibroblasts & capsule cells, whereas progenitor cells were less abundant compared to the *KCNJ5*‐mutated APA (Figure S1C, Supporting Information). These findings support the concept that distinct driver mutations may influence the tumor microenvironment by shaping the cellular composition in APA.^[^ ^30^, ^31^, ^32^ ^]^

### 2.2 Immunosuppressive Tumor Microenvironment in APAs

**Figure 2:**
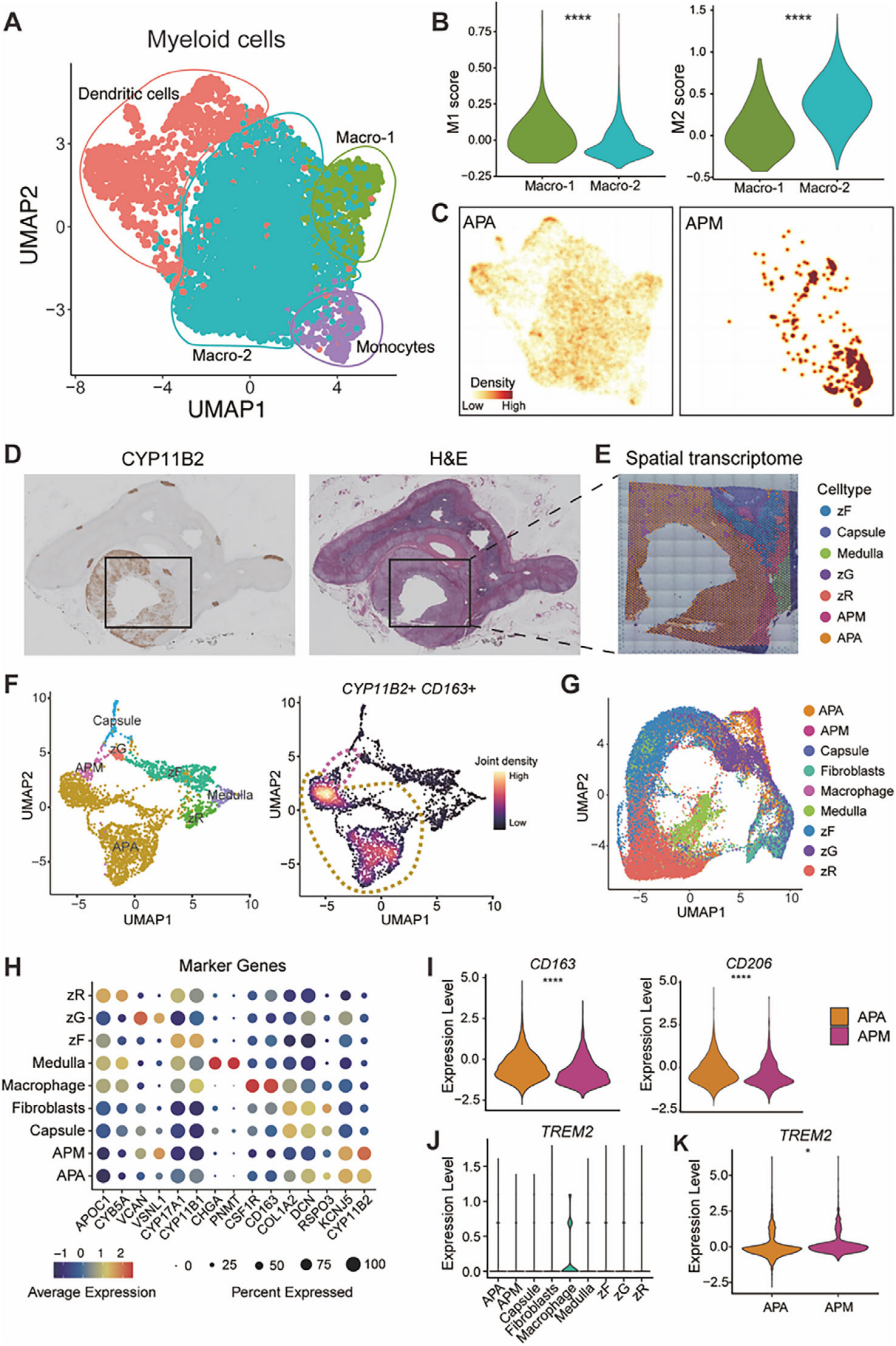
Characterization of the immune microenvironment in APAs. A) UMAP plot demonstrating myeloid subpopulations. B) Violin plots showing the average expression levels of M1 and M2 macrophage signature gene panels across different macrophage populations. Statistical significance was determined using a two‐sided Wilcoxon rank‐sum test. C) Changes in the composition of the myeloid compartment between APA and APM visualized as cell density on the joint embedding. D) CYP11B2 immunohistochemistry staining (left) and H&E staining (right) of one spatial transcriptomics section. E) Spatial cluster distribution in one section. F) UMAP plot depicting the 7 spatial cell types (left) and co‐expression of *CYP11B2* with *CD163* in the same section. G) UMAP visualization of 10 major spatial cell types integrated across 12 tissue sections using Spatial RNA‐seq Technology. H) Bubble plot of representative cell‐type specific markers across all cell types. I) Violin plots illustrating the expression of *CD163 and CD206* in APA and APM cell types. Statistical significance was determined using a two‐sided Wilcoxon rank‐sum test. J) Violin plot displaying *TREM2* expression across all cell types. K) Violin plot showing expression of *TREM* in the APA and APM cell types. Statistical significance was determined using a two‐sided Wilcoxon rank‐sum test. APA, aldosterone‐producing adenoma; APM, aldosterone‐producing micronodule; H&E, hematoxylin and eosin; zF, zona fasciculata; zG, zona glomerulosa; zR, zona reticularis; ^*^ *P* < 0.05 and ^****^ *P* < 0.0001.

Immune cell infiltration is a common feature in various tumor types, including APAs,^[^ ^29^, ^33^ ^]^ with macrophages especially prevalent in endocrine organs like the adrenal gland.^[^ ^34^ ^]^ We identified eight distinct subclusters of myeloid cells (Figure S2A, Supporting Information), the top five up‐ and down‐regulated genes in each subcluster are shown in a volcano plot (Figure S2B, Supporting Information). Subpopulation analysis of myeloid cells, based on canonical markers, revealed distinct cellular populations within the tumor microenvironment: one group of monocytes, one group of dendritic cells, and two distinct macrophage populations (Macro‐1 and Macro‐2) (**Figure** **2**A; Figure S2C, Supporting Information). Macro‐2 showed a significantly increased M2 macrophage signature score compared to Macro‐1 (Figure 2B) and expressed characteristic M2 macrophage marker genes (*CD209*, *EGR2*, and *HMOX1*) (Figure S2D, Supporting Information). This suggests that Macro‐2 macrophages likely play an immunosuppressive role and potentially support tumor growth. In contrast, Macro‐1 demonstrated a significantly higher M1 signature score relative to Macro‐2 (Figure 2B) and expressed elevated levels of *IL15* and *CD86* (Figure S2D, Supporting Information), indicative of a pro‐inflammatory state.

Within the myeloid subpopulation, the proportions of dendritic cells and macrophages were significantly elevated in the APA fraction compared to the APM cell type, whilst monocytes were markedly reduced (Figure 2C; Figure S2E, Supporting Information). Compositional data analysis corroborated these findings, revealing a higher abundance of M2 macrophages in APA than in APM, suggesting an immunosuppressive tumor microenvironment at the local site of APA (Figure S2F, Supporting Information).

The spatial distribution of myeloid cells in both APMs and APAs was investigated using a PA‐adrenal sample section. In this section, APA and APM areas were identified though morphology staining with hematoxylin and eosin (H&E) and CYP11B2 immunohistochemistry for specific identification of aldosterone‐producing cells (Figure 2D). Spatial transcriptomics data were obtained using the Visium formalin‐fixed paraffin embedded (FFPE) pipeline. Barcoded spots were then histologically annotated based on H&E‐stained and CYP11B2‐immunostained images, combined with Seurat‐based clustering analysis (Figure 2E).

Our analysis revealed a higher number of co‐infiltrating *CD163* ^+^ and *CYP11B2* ^+^ cells within the APA tumor microenvironment (Figure 2F), consistent with our findings at the sc/snRNA level. The generalizability of this finding was validated by integrating spatial transcriptomics data from all 12 tissue specimens (Figures S3 and S4 and Table S5, Supporting Information). For this, we deconvolved the spatial transcriptomics data using UMAP for dimension reduction and to visualize the basic structural transcriptomic landscapes corresponding to histologically discernible features (Figure 2G). Subsequently, we assigned barcoded spots to known cell lineages based on specific marker genes (Figure 2H). This analysis demonstrated that APA cells expressed significantly higher levels of *CD163* and *CD206* than APM cells (Figure 2I). High CD206 expression was observed across all genotypes, including *KCNJ5*‐, *CACNA1D*‐, *ATP1A1*‐ and *ATP2B3*‐mutated APAs, suggesting that M2 macrophage involvement is a common feature irrespective of mutational background (Figure S5A,B, Supporting Information). Among *KCNJ5*‐mutated APAs, all female patients exhibited high CD206 expression, whereas male patients displayed more heterogeneous expression patterns (Figure S5C, Supporting Information). However, this difference was not statistically significant (Fisher's exact test, *P* = 0.553), indicating no strong association between CD206 status and patient sex.

Previous research has shown that *TREM2* co‐localizes with tissue macrophages in the adrenal cortex, and macrophage *TREM2* deficiency increases steroidogenesis in adrenocortical cells.^[^ ^35^ ^]^ Here, we found that *TREM2* is exclusively expressed in the macrophage population and that *TREM2* expression was significantly decreased in APAs compared to APMs (Figure 2J,K).

### 2.3 Intercellular Communication Networks and Key Signaling Pathways in APAs

**Figure 3:**
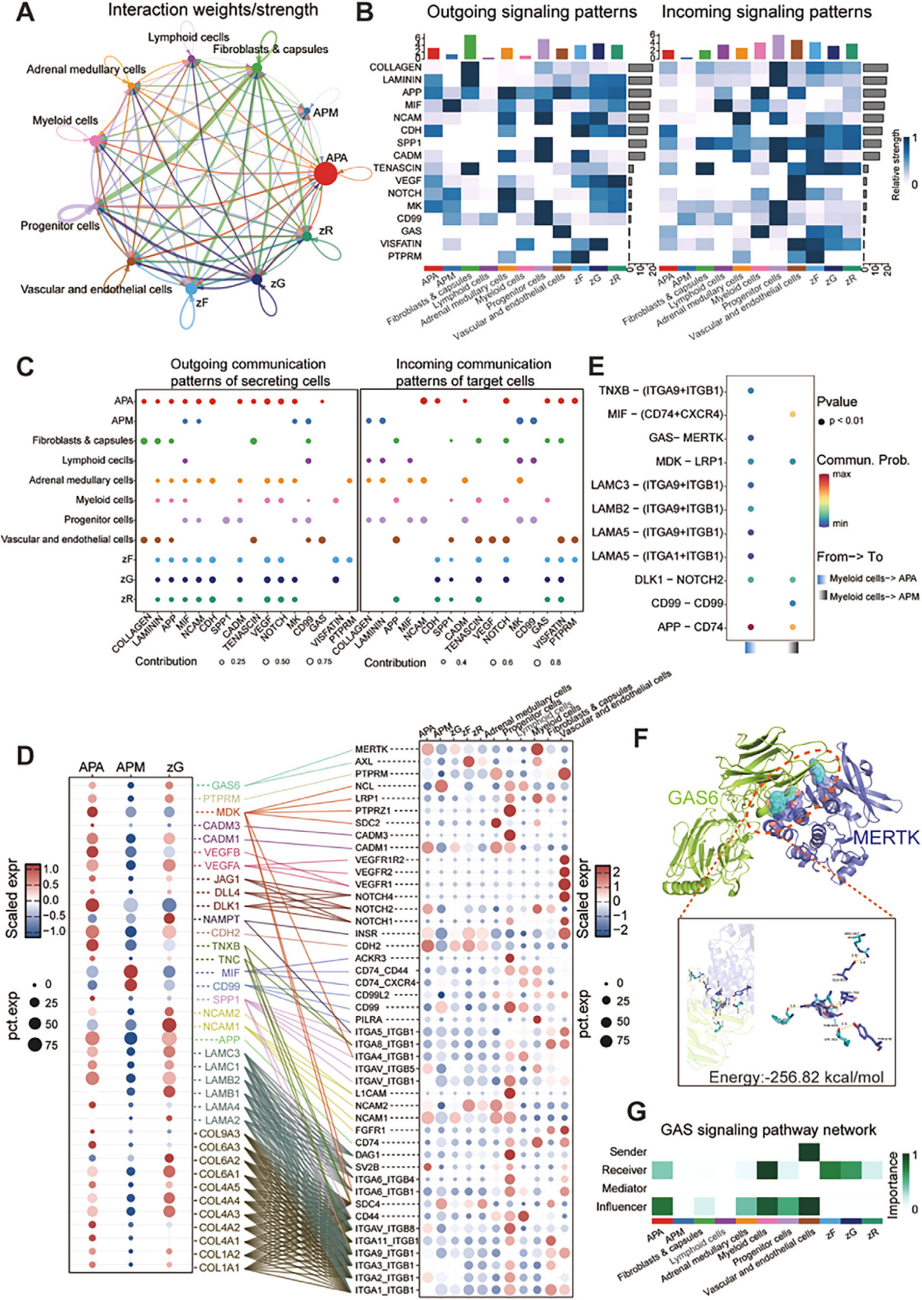
Complex cell–cell communication networks in APA. A) Weighted total interaction strength. Line thickness denotes interaction strength, while color corresponds to different cell types. B) Heatmap showing the strength of outgoing and incoming interaction events between cell types. Bars at the top of the heatmaps summarize the total intensity of outgoing and incoming events per cell type, which are indicated at the bottom of the heatmaps. Rows are ordered by contributing signaling pathways. Color intensity represents interaction strength. C) Bubble plots depicting intercellular communication patterns among different cell types. Left: outgoing contributions; Right: incoming contributions. Bubble color represents distinct cell types; bubble size indicates the strength of communication. D) Bubble plots showing gene expression levels of receptor‐ligand pairs involved in interactions between cell types. Bubble color indicates average relative expression; bubble size indicates percentage of relative expression. E) Communication probabilities of important ligand‐receptor pairs from myeloid cells to individual APA and APM cells, with bubble color indicating communication probability and size representing the *P* values. F) Molecular docking analysis showing the predicted 3D structure (top) and intermolecular interactions (bottom) of GAS6 binding to MERTK. G) Function of the GAS signaling pathway in cellular interaction. APA, aldosterone‐producing adenoma; APM, aldosterone‐producing micronodule; zF, zona fasciculata; zG, zona glomerulosa; zR, zona reticularis.

Cell–cell communication analysis using CellChat^[^ ^36^ ^]^ revealed that the APA cell cluster exhibited strong interaction weights and robust signaling strength of incoming and/or outgoing cell communications (**Figure** **3**A,B). APAs exhibited the most pronounced cell–cell contact phenotype among the 11 annotated cell types, as determined by analysis of three modes of cell–cell communication (cell–cell contact, extracellular matrix‐receptor, and secreted signaling) from ligand‐receptor pair interactions (Figure S6A, Supporting Information). Notably, genotype‐stratified CellChat analysis revealed that *KCNJ5*‐mutated APA samples exhibited enhanced cell–cell contact interactions, particularly through Cadherin (CDH) signaling pathways, in communication with other cell types such as zG cells (Figure S6B, Supporting Information).

The captured ligand‐receptor network displayed functional diversity, which can be linked to distinct signaling pathways observed across different cell types (Figure S6C,D, Supporting Information). The NCAM, CDH, CADM, and PTPRM pathways contributed to APA cell–cell communication, with APA cells expressing higher levels of transcription of the corresponding ligands (NCAM1, NCAM2, CDH2, CADM1, and PTPRM) compared to APM cells (Figure 3C,D *)*. Additionally, signaling through NCAM, CDH, CADM, and PTPRM was active in cell–cell communication between APA cells and in interactions with other cell types annotated in the adrenal cortex, particularly zG and zona fasciculata cells. In contrast, these signaling pathways were absent or diminished in interactions involving APM cells (Figure S6E, Supporting Information).

Given the previous implication of myeloid cell populations, particularly macrophages, in APA development, we assessed cell–cell communication between myeloid cells and APA cells and between myeloid cells and APM cells by ligand‐receptor analysis. The communication probability mediated by the GAS6‐MERTK pair was significantly higher from myeloid cells to APA cells compared to APM cells (Figure 3E). GAS6/MERTK signaling has been shown to promote an M2‐like phenotype in macrophages, leading to an immunosuppressive microenvironment.^[^ ^37^, ^38^ ^]^ While the macrophage‐mediated role of MERTK in APA is unexplored, its expression was upregulated in human myeloid‐derived macrophages following treatment with aldosterone, supporting MERTK expression in anti‐inflammatory M2‐like macrophages.^[^ ^37^ ^]^ Molecular docking confirmed the potential for cell–cell interactions via the GAS6‐MERTK pair, indicating a strong and favorable binding interaction with an energy of ‐256.82 kcal mol^−1^ (Figure 3F). Further findings highlighted the central role of the GAS signaling network in APA, acting as both a receiver and an influencer (Figure 3G). In contrast, the MIF‐(CD74+CXCR4) pairs were specifically enriched in myeloid‐APM cell communications (Figure 3E), potentially contributing to more comprehensive functions in APM across sender, receiver, mediator, and influencer roles (Figure S6F, Supporting Information). This cell–cell communication analysis highlights the heterogeneity of signaling pathways and the complexity of intercellular ligand‐receptor interactions in different cellular contexts of aldosterone‐producing cells, specifically in APMs and APAs.

### 2.4 Gene Regulatory Networks Reveal Key Transcription Factors in APA and APM Cells

**Figure 4:**
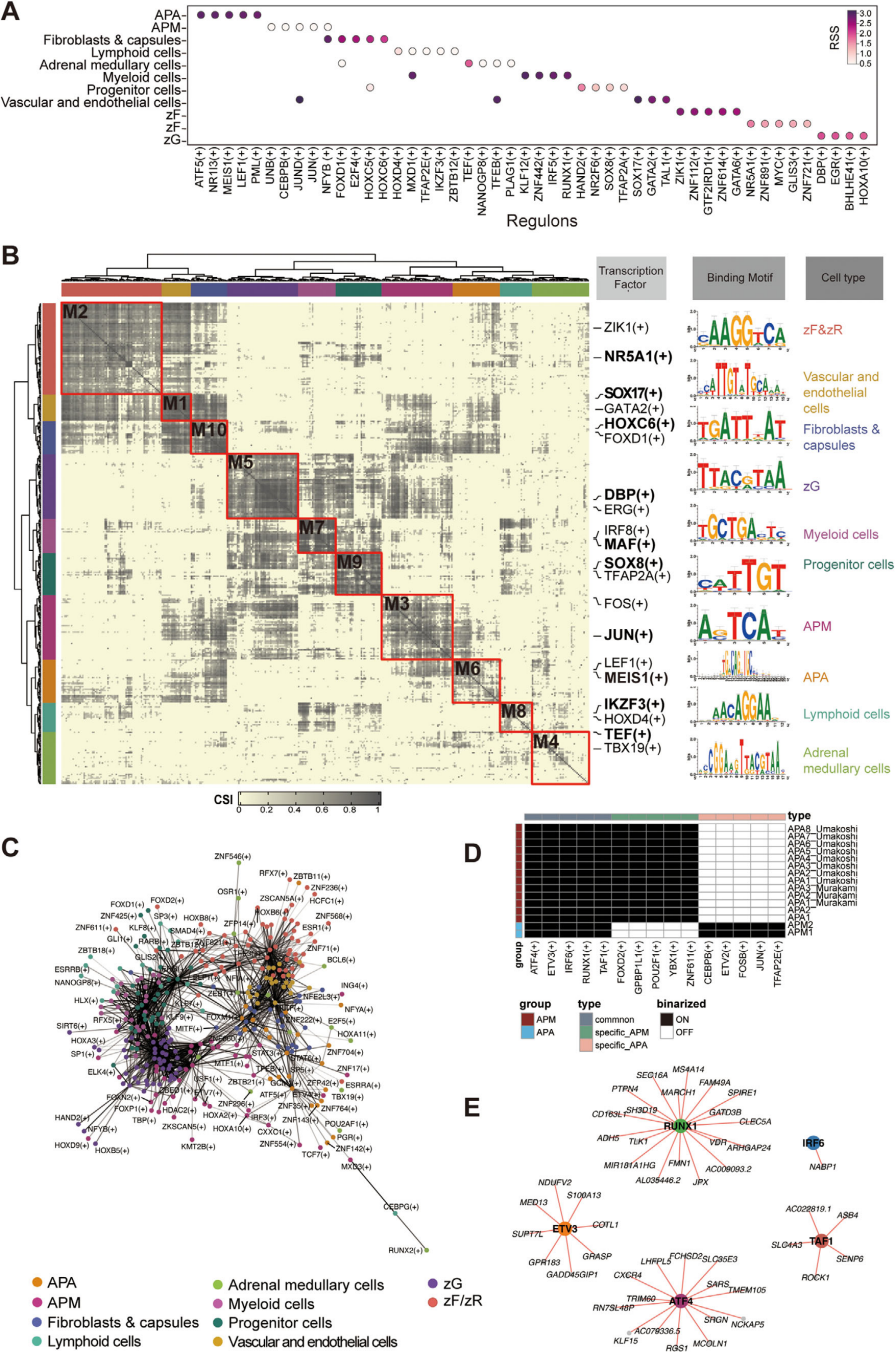
Cell‐type‐specific regulon activity analysis and identification of combinatorial regulon modules. A) Rank for regulons in each cell type based on regulon specificity score (RSS). The “regulon” refers to the regulatory network of transcription factors (TFs) and their target genes. B) Combinatorial regulation modules are identified based on regulatory connection specificity index (CSI) matrices, along with representative transcription factors, corresponding binding motifs, and associated cell types. C) Interaction network for the combinatorial regulation modules based on similarity of regulon activities. D) Heatmap of regulon activity analysed by single‐cell regulatory network inference and clustering with default thresholds for binarization. “On” indicates active regulons; “Off” indicates inactive regulons. E) Regulatory networks consisting of shared transcription factors and their target genes. APA, aldosterone‐producing adenoma; APM, aldosterone‐producing micronodule; zF, zona fasciculata; zG, zona glomerulosa; zR, zona reticularis.

To investigate master regulators governing various cell clusters, we constructed gene regulatory networks using SCENIC (single‐cell regulatory network inference and clustering) analysis. Regulon specificity scores identified and ranked top regulons active in specific cell types (**Figure** **4**A). Among the transcription factors (TFs) with the highest specificity in APA cells, LEF1 (+) and MEIS1 (+) were of particular interest due to their previously reported roles in adrenocortical disease and their association with adrenocortical cell proliferation and steroidogenic gene regulation.^[^ ^39^, ^40^ ^]^ Re‐clustering regulons using the connection specificity index identified 379 regulons in 10 distinct modules (Figure 4B; Table S6, Supporting Information). Module 7 contained regulons specifically activated in myeloid cells (e.g., *MAF*, *IRF8*), while Module 6 comprised APA‐specific regulons like *LEF1* and *MEIS1*. A TF‐to‐TF network based on the detected TFs revealed a complex structure across cell types (Figure 4C).

To elucidate gene regulatory network differences between APA and APM cells, we implemented a scoring strategy transforming regulons into a binary matrix reflecting TF activation status (Figure 4D; Table S7, Supporting Information). Other than cell‐specific TF transcripts, others were significantly represented in both cell states (*ETV3, ATF4*, *RUNX1*, *IRF6* and *TAF1*), suggesting potential roles in sustaining the adenoma developmental programme throughout disease progression. The regulatory network of these shared TFs and their target genes was depicted based on weight values (Figure 4E).

### 2.5 Pseudotime Trajectory Analysis Indicates APMs as Precursors to APA

To elucidate the developmental lineage relationships among the zG, APM, and APA cell subgroups, we performed pseudotime trajectory analysis using the integrated sc/snRNA‐seq dataset that incorporated 5 peritumoral adrenal tissues adjacent to APA derived from cases with *KCNJ5* or *ATP2B3* mutations (GSE210381).^[^ ^41^ ^]^ This result revealed that the cell subgroups originated from an initial point in the trajectory, diverging at the first branch point and further differentiating into two distinct branches (Figure S7A, Supporting Information). APM and most zG cells were in the early stages of the pseudotime trajectory, while the APA cells represented two terminal states with no potential to differentiate into other lineages (Figure S7B, Supporting Information). Individual pseudotime analyses stratified by *KCNJ5*, *ATP1A1*, or *ATP2B3* mutation indicated that APA cells exhibited broadly consistent distributions along the pseudotime continuum originating from similar zG‐ and APM‐derived cells (Figure S7C, Supporting Information), supporting the developmental model derived from the integrated analysis.

**Figure 5:**
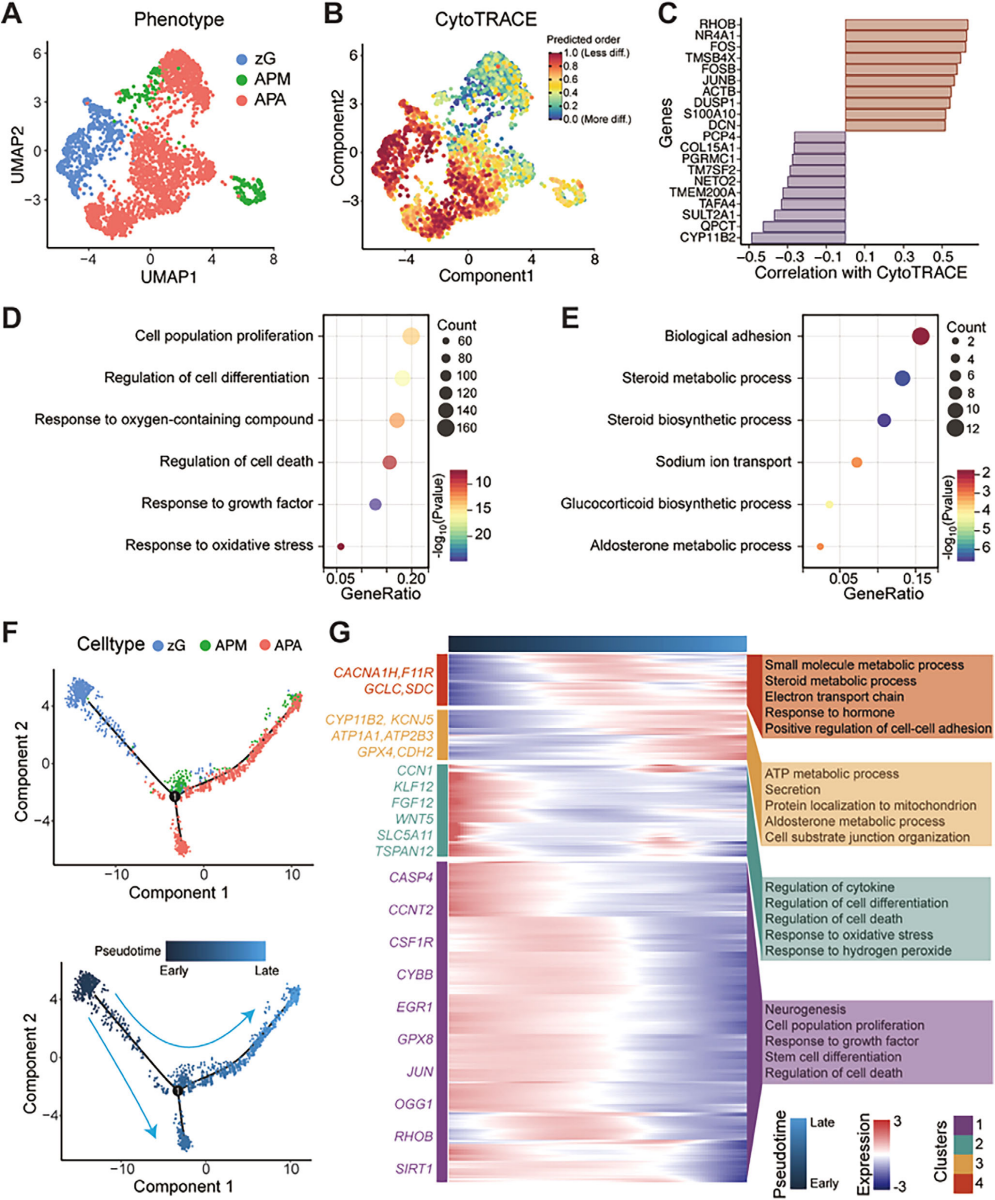
Pseudotime analysis of zG, APM, and APA cells in three spatial transcriptome sections. A) UMAP plot illustrating the distribution of zG, APM, and APA cells at the integrated statical transcriptome (ST) level. B) UMAP plot showing cell differentiation status using CytoTRACE. Color scale from dark green (low stemness) to dark red (high stemness) indicates differentiation levels. C) Bar plot showing genes correlated with the highest and lowest differentiation levels based on their correlation with CytoTRACE scores. D,E) Functional annotation of regulatory genes driving heightened cell stemness (D) and reduced cell stemness (E). F) Reconstructed pseudotime evolution trajectory of zG, APM, and APA cells inferred by Monocle2. The trajectory plot showing the three cell types assigned by different colors (top) and the direction of developmental time points arranged by pseudotime (bottom). The arrows indicate the likely course of evolution within the trajectory. G) **Heatmap of gene dynamics** along the reconstructed cell trajectories, with columns representing cells and rows denoting genes. Genes are ranked and divided into four clusters based on expression trends on the left, accompanied by corresponding functional annotations on the right. APA, aldosterone‐producing adenoma; APM, aldosterone‐producing micronodule; zG, zona glomerulosa.

Given the shared origin of zG and APM cells at the sc/snRNA‐seq level, identifying the true differentiation starting point proved challenging. This prompted us to utilize spatial transcriptomics data from three slides of patient #4 carrying an APA *KCNJ5* mutation for further analysis (Figure S8A,B, Supporting Information). We first employed a UMAP plot to visualize the distribution of the three cell subgroups (zG, APM and APA) in the integrated data (**Figure** **5**A). To further elucidate the differentiation and developmental relationships among these subgroups at the spatial transcriptomics level, we conducted a cell differentiation analysis using CytoTRACE. The results indicated that zG cells exhibited the highest degree of stemness, highlighting their primitive nature in the differentiation hierarchy (Figure 5B; Table S8, Supporting Information).

We also identified genes with the strongest and weakest associations with the differentiation process based on CytoTRACE scores (Figure 5C). Subsequently, we performed corresponding Gene Ontology (GO) enrichment analyses for the two groups based on their positive and negative correlations. Genes involved in the oxidative stress response (GO:0006979), such as *RHOB, FOS, DUSP1*, and *JUNB*, were associated with less differentiated zG cells (Figure 5C,D). In contrast, genes associated with aldosterone metabolism (GO:0032341), including *CYP11B2* and *CACNA1H*, as well as biological adhesion molecules (GO:0007155) like *CDH2*, *CCN3*, and *CXCL12*, showed higher correlation with terminally differentiated cells (Figure 5C,E). These findings suggest that zG cells may have greater potential to differentiate into other subpopulations under oxidative stress conditions.

Additionally, we employed Monocle2 to comprehensively investigate the origins and developmental pathways leading to adenoma cells. Consistent with CytoTRACE findings, zG cells were positioned at the beginning of the pseudotime series, while APA cells represented two terminal states with no potential for further differentiation (Figure 5F). APM cells were located at the branching point and progressed alongside the development of one of the APA terminal states, suggesting a stepwise trajectory from zG cells to APMs and then to APAs, in addition to a direct transition from zG cells to APAs. This dual‑trajectory pattern was further supported by an integrated spatial transcriptomics analysis of three *KCNJ5*‐mutated samples (patient #4 plus two additional cases, patients #5 and #6). As patients #5 and #6 contained too few captured APA cells for separate trajectory reconstruction but did include zG and APM cells, these could be incorporated into the integrated pseudotime analysis, which consistently recapitulated both direct and indirect routes of APA formation (Figure S8C–H, Supporting Information). In contrast, spatial analysis of three APAs without a *KCNJ5* mutation‐ one *ATP1A1*‐mutated (patient #1), one *CACNA1D*‐mutated (patient #3), and one without a detectable driver mutation (patient #2)‐ revealed a different developmental pattern. Although individual samples had limited zG and APM capture, integrated analysis across these APAs demonstrated a single dominant trajectory from zG through APM to APA, without evidence of a direct zG to APA path (Figure S9, Supporting Information). These results suggest that the direct differentiation route of zG to APA may be a specific feature of *KCNJ5*‐mutated APAs, whereas other genotypes may preferentially follow a stepwise progression through APMs.

To explore the molecular determinants of different cell subgroups, we conducted gene expression analysis along the pseudotime trajectory in *KCNJ5*‐mutated APA, identifying four distinct gene clusters based on their expression patterns (Figure 5G; Table S9, Supporting Information). Analysis of gene alterations along the trajectory revealed that most genes in clusters 1 and 2, including *JUN, RHOB, KLF12*, and *CCN1*, exhibited a monotonically decreasing expression across pseudotime. These genes were enriched in biological processes related to oxidative stress response, regulation of cell death, and cell differentiation, suggesting a link to less differentiated cells. In contrast, canonical mutation markers associated with APA, such as *KCNJ5, ATP1A1*, and *ATP2B3*, along with *CYP11B2* in gene cluster 3, showed a gradual increase in expression across pseudotime, aligning with the tumorigenic process and progression of APA. For gene cluster 4, we observed non‐monotonic expression trends with high levels of *CACNA1H*, *F11R*, *GCLC*, and *SDC*. The enriched terms for these genes included steroid metabolic processes and positive regulation of cell–cell adhesion, indicating their potential role in maintaining specific cellular functions.

### 2.6 Dynamic Regulatory of Oxidative Stress and Intercellular Adhesion during APA Progression

**Figure 6:**
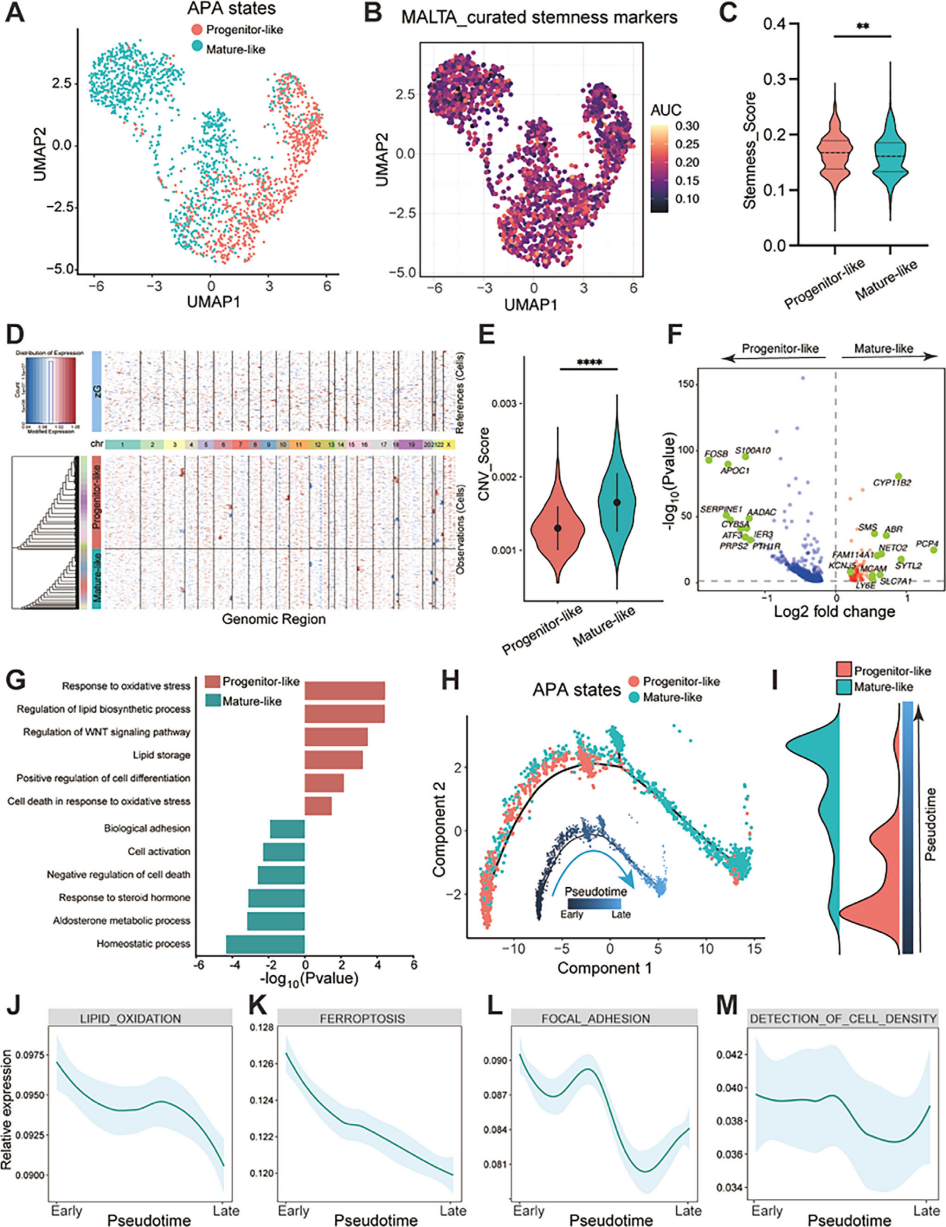
Trajectory analysis of biological process during APA progression. A) UMAP plot illustrating two distinct APA cell differentiation states based on the CytoTRACE score: progenitor‐like (high differentiation potential, CytoTRACE >0.5) and mature (low differentiation potential, CytoTRACE ≤0.5) states. B) UMAP plot showing the distribution of cell stemness scored each cell by AUCell. C) Violin plot comparing stemness scores between progenitor‐like and mature APA cell states. Statistical significance was determined using a two‐sided Wilcoxon rank‐sum test. D) Heatmap showing large‐scale copy number variation (CNV) profile of each APA cell. Red and blue colors represent high and low CNV levels, respectively. zG cells are defined as reference cells. E) Violin plot showing the CNV scores between progenitor‐like and mature APA cell states. Statistical significance was determined using a two‐sided Wilcoxon rank‐sum test. F) Volcano plot showing differentially expressed genes between progenitor‐like and mature APA cell states, with gene colors according to cell subtypes. G) Functional annotation analysis depicting the Gene Ontology (GO) biological process enriched in progenitor‐like and mature APA cell states, with process colors according to cell states. H) Reconstructed pseudotime evolution trajectory of APA cells inferred by Monocle2. The trajectory plot showing the two APA states assigned by different colours (top) and the direction of developmental time point arranged by pseudotime (bottom). The arrow indicating the likely course of evolution within the trajectory. I Cell density variation of APA states across pseudotime, with density colors according to states. J–M) Expression dynamics of key biological processes during APA progression, including lipid oxidation (J), ferroptosis (K), focal adhesion (L), and detection of cell density (M), showing changes over pseudotime. ^**^ *P* < 0.01 and ^****^ *P* < 0.0001. APA, aldosterone‐producing adenoma.

We further investigated the evolution of oxidative stress and intercellular adhesion during APA progression by analyzing APA cells at different stages of differentiation. Two distinct cellular differentiated states with different developmental potentials were categorized based on their CytoTRACE scores: a progenitor‐like state (CytoTRACE > 0.5) and a mature state (CytoTRACE ≤ 0.5) (**Figure** **6**A). Higher CytoTRACE scores indicate a less differentiated state with greater developmental potential, suggesting proximity to the developmental origin. To assess the stemness of APA cells in mature and progenitor‐like states, we applied AUCell to quantify stemness in these cell states. The results demonstrated that mature APA cells exhibited lower stemness compared to progenitor‐like APA cells (Figure 6B,C). We then inferred somatic large‐scale chromosomal copy number variations (CNVs) and calculated CNV scores to compare tumor characteristics between mature and progenitor‐like APA cell states, using zG cells as a reference. As expected, the mature APA cell group was characterized by unique CNV amplifications on various chromosomes, particularly chromosomes 16, with significantly higher CNV scores than the progenitor‐like APA states (Figure 6D,E).

We performed differential gene expression (DEG) and GO term analyses on the mature and progenitor‐like APA cells, revealing that *CYP11B2*, *NETO2*, *PCP4*, and *KCNJ5* were more highly expressed in mature APA cells compared to their progenitor‐like counterparts (Figure 6F; Table S10, Supporting Information). These findings further supported the accuracy of our classification of APA cell states. GO analysis showed that mature APA cells were primarily associated with responses to steroid hormones and biological adhesion, whereas progenitor‐like APA cells were enriched for processes related to lipid biosynthesis regulation and cell death in response to oxidative stress (Figure 6G). Together, these results suggest that progenitor‐like APA cells experience higher oxidative stress than mature APA cells.

To elucidate the maturation process of APA cells, we performed a trajectory analysis within this population. Notably, the progenitor‐like APA cells were identified as the origin of cell differentiation, with the terminal branches predominantly populated by mature APA cells (Figure 6H), as confirmed by the cell density map (Figure 6I). We analysed the changes in biological processes involved in the transition of APA states. Results showed that oxidative stress‐related processes, such as lipid oxidation, peroxisome function, and peroxisome proliferator‐activated receptor alpha activity, gradually decreased along the pseudotime axis (Figure 6J; Figure S10A, Supporting Information). To further validate the transcriptional signatures identified along the pseudotime trajectory, we examined oxidative stress by IHC staining for malondialdehyde (MDA), a marker of lipid peroxidation. Consistent with the pseudotime‐based inference, MDA immunostaining was generally decreased in APA tumor regions compared to the adjacent adrenal cortex, suggesting a reduced oxidative stress state in APA tumor cells.^[^ ^42^ ^]^ We next explored whether this oxidative phenotype varied across different APA genotypes. MDA staining levels were compared among APA samples with known mutations in *KCNJ5* (n = 21), *CACNA1D* (n = 5), *ATP1A1* (n = 4) or *ATP2B3* (n = 3) (Figure S11A, Supporting Information). A statistically significant difference in MDA distribution was observed among these subtypes (P = 0.007, Fisher's exact test), with *KCNJ5*‐mutated APAs more frequently exhibiting reduced MDA levels (Figure S11B, Supporting Information). These results support a genotype‐specific redox state, in line with the observed transcriptional downregulation of oxidative stress‐related pathways.^[^ ^31^, ^42^ ^]^ Additionally, we assessed whether oxidative stress patterns differed by sex. Because *ATP1A1*‐, *ATP2B3*‐, and *CACNA1D*‐mutated APAs are relatively uncommon in our cohort and occur mainly in males, we focused on *KCNJ5*‐mutated cases, which, while more frequent in females, are the most prevalent overall and allow for representation of both sexes.^[^ ^43^ ^]^ Among these, no significant sex‐based differences in MDA immunostaining were found, suggesting that sex‐related factors may not substantially influence oxidative stress in APA (Figure S11C, Supporting Information).

Given that lipid peroxidation is a key characteristic of ferroptosis, we calculated the expression of ferroptosis‐related genes and observed a similar decreasing trend (Figure 6K). Furthermore, ferritin heavy chain 1 (*FTH1*) mRNA levels, crucial for ferroptosis defence by sequestering redox‐active iron,^[^ ^44^ ^]^ were higher in the late stage of pseudotime (Figure S10B, Supporting Information). Conversely, transferrin receptor protein 1 (*TfR1*), key to importing iron from the extracellular environment,^[^ ^45^ ^]^ was downregulated along APA progression (Figure S10C, Supporting Information). The expression of these two marker genes differed significantly between the progenitor‐like and mature APA states (Figure S10B,C, Supporting Information). To explore the potential mechanistic implications of non‐genetic determinants in ferroptosis, we investigated changes in focal adhesion, observing diverse patterns (Figure 6L). Cadherin 2 (*CDH2*), a transmembrane molecule found in several types of cell–cell contacts, plays a role in ferroptosis resistance; its depletion increases susceptibility to ferroptotic death.^[^ ^46^ ^]^ In the progenitor‐like state of APA progression, low *CDH2* expression might be associated with increased ferroptosis susceptibility (Figure S10B,C, Supporting Information). Cell density has been reported as an important regulator of focal adhesion kinase localization.^[^ ^47^ ^]^ Our analysis of cell density revealed a fluctuating pattern, characterized by a moderate decline followed by a recovery (Figure 6M). Collectively, the APA developmental trajectory revealed a dynamic regulatory environment involving oxidative stress (including mechanisms leading to cell death by ferroptosis) and focal adhesion (influenced by cell density).

### 2.7 The Hippo Pathway Effector *TAZ* Regulates Ferroptosis in Human HAC15 Cells

**Figure 7:**
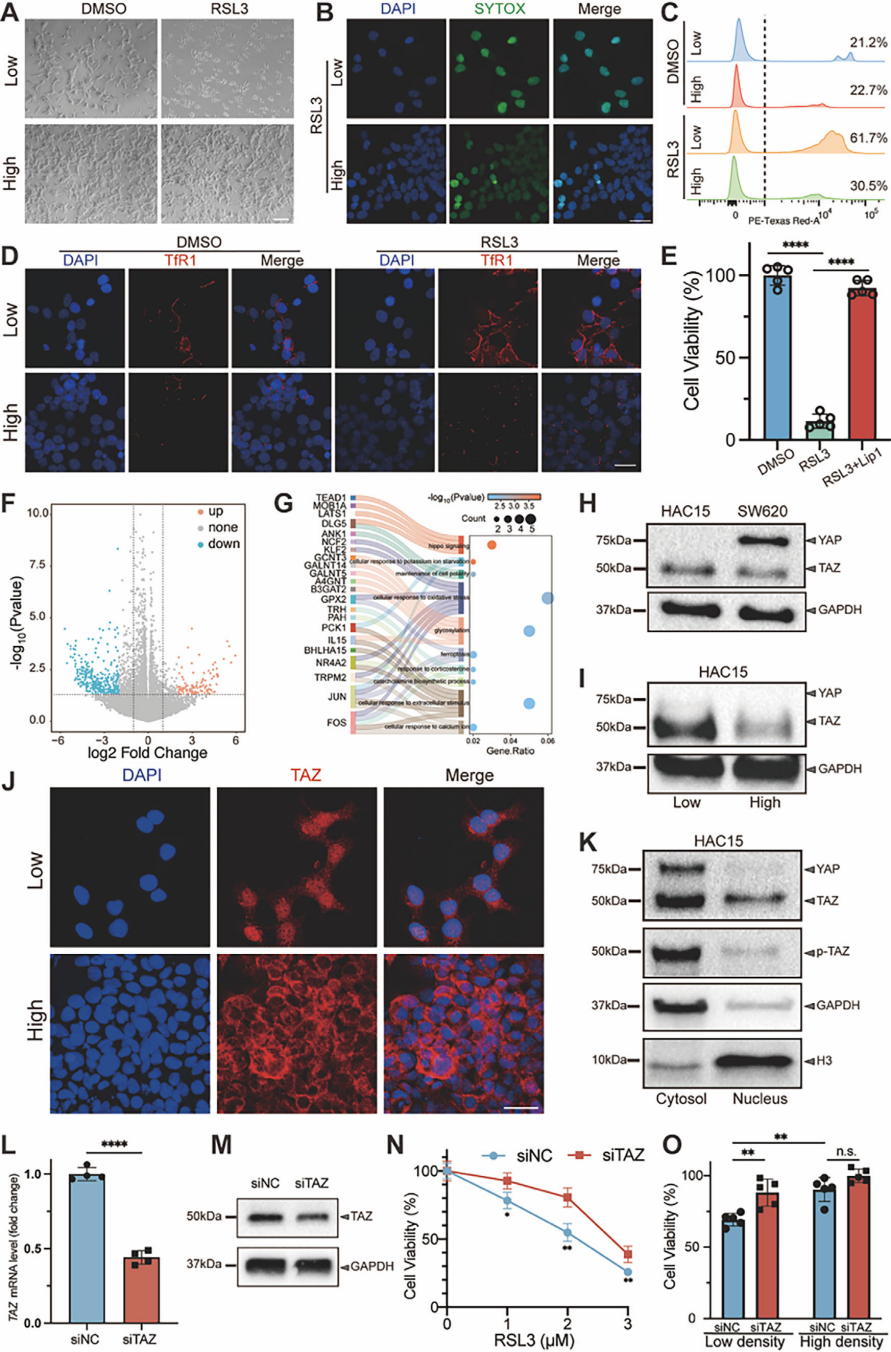
Hippo pathway effectors and ferroptosis in human adrenal cells. A) Bright‐field images of HAC15 cells cultured at low or high densities treated with DMSO or 4 µM RSL3. Representative data of at least three independent experiments. Scale bar, 200 µm. B,C) Cell death analysis of HAC15 cells cultured at low or high densities treated with DMSO or 4 µM RSL3 by immunofluorescence (B) and flow cytometry (C). Representative data of at least three independent experiments. Scale bar, 100 µm. D) TfR1 immunofluorescence in HAC15 cells cultured at low or high densities treated with DMSO or 4 µM RSL3. Representative data of three independent experiments. Scale bar, 100 µm. E) Cell viability of HAC15 determined by WST‐1 assay following treatment with 4 µM RSL3 or Lip‐1 + 4 µM RLS3 for 4 h (n = 5 independent experiments). Statistical analysis was performed using two‐way ANOVA. F) Volcano plot showing differentially expressed genes of HAC15 cells treated with 4 µM RSL3 cultured for 3 h at low versus high cell densities. G) Gene Ontology (GO) biological process of differential gene expression between HAC15 cells treated with 4 µM RSL3 at low and high cell densities. H) Western blot analysis of YAP and TAZ expression in HAC15 cells and in the colon cancer cell line, SW620. I) Western blot analysis of YAP and TAZ in HAC15 cells cultured at low or high densities. J) Immunofluorescence images of TAZ in HAC15 cells cultured at low or high densities. TAZ was localized in the cytoplasm at high cell density and in the nucleus at low density. Representative data of at least three independent experiments. Scale bar, 100 µm. K) YAP/TAZ and phospho‐TAZ (Ser89) expression in cytoplasm and/or nucleus of HAC15 cells. H3, nuclear marker; GAPDH, cytosolic marker. L) The mRNA expression of *TAZ* in HAC15 cells after *TAZ* gene silencing (n = 4 independent experiments). Statistical analysis was performed using an unpaired two‐tailed Student's *t*‐test. M) The protein of TAZ in HAC15 cells after *TAZ* gene silencing. N) WST‐1 assay of siTAZ with the indicated concentrations of RSL3 (n = 4 independent experiments). Data are presented as mean ± SEM. Statistical analysis was performed using two‐way ANOVA. O) HAC15 cells cultured in low/high densities when treated with siNC or siTAZ for 24 h. Cell viability was determined by WST‐1 assay after 24 h treatment with 4 µM RSL3 (n = 5 independent experiments). Data are presented as mean ± SEM. Statistical analysis was performed using two‐way ANOVA. DMSO, dimethyl sulfoxide; RSL3, 1S,3R‐RSL3, is a glutathione peroxidase 4 (GPX4) inhibitor. Lip‐1, liproxstatin‐1 hydrochloride is a potent ferroptosis inhibitor. siNC, siRNA negative control; siTAZ, siRNA targeting *TAZ*. ^*^ *P* < 0.05, ^**^ *P* < 0.01 and ^****^ *P* < 0.0001. n.s., no significance.

The zG‐to‐APM‐to‐APA trajectory and APA progression stages reflect macro‐level changes in tissue architecture and cell density, accompanied by ferroptosis dynamics. To validate this relationship at a micro‐level, we conducted in vitro experiments to examine ferroptosis sensitivity under varying cell density conditions. Our results showed that RSL3 sensitivity is influenced by cell density. When HAC15 cells were grown at low density, they were highly sensitive to RSL3‐induced cell death, as evidenced by morphological changes such as cell rounding observed under light microscopy (**Figure** **7**A). In contrast, cells grown at high density exhibited reduced RSL‐sensitivity. This cell density‐regulated sensitivity was further confirmed using cell death assays with SYTOX Green and propidium iodide staining (Figure 7B,C). Immunofluorescence analysis revealed increased TfR1 localization in the plasma membranes of RSL3‐treated cells compared to controls under low‐density conditions (Figure 7D). To rule out the possibility that cell density alters the mode of RSL3‐induced cell death, we found that the ferroptosis inhibitor liproxstatin‐1 rescued RSL3‐induced cell death in low‐density cultures (Figure 7E). These findings indicate that cell density regulates ferroptosis sensitivity in HAC15 cells. To investigate the underlying mechanisms, we compared the transcriptomes of HAC15 cells treated with RSL3 under low‐ and high‐cell density conditions (Figure 7F). GO analysis revealed that the differentially expressed genes were associated with the Hippo pathway, cellular response to oxidative stress, and ferroptosis (Figure 7G).

The Hippo pathway effectors YAP and TAZ function as molecular sensors, mediating cell density‐dependent responses through phosphorylation‐mediated regulation and dynamic subcellular localization.^[^ ^48^, ^49^ ^]^ To investigate the role of *YAP*/*TAZ* in ferroptosis, we first assessed the expression of YAP and TAZ proteins in the HAC15 cell line. We found that TAZ was the predominant coactivator in HAC15 cells, while YAP was not expressed, in contrast to the predominant expression of YAP in SW620 cells (Figure 7H). We compared TAZ protein levels across different cell densities and observed that higher cell density correlated with lower TAZ expression (Figure 7I). We demonstrated that in HAC15 cells, TAZ exhibits cell density‐dependent subcellular localization, predominantly residing in the cytoplasm at high densities while translocating to the nucleus in low‐density cultures (Figure 7J). Cytoplasmic and nuclear fractionation analyses confirmed that nuclear TAZ accumulation correlates with reduced phosphorylation levels (Figure 7K), suggesting that TAZ dephosphorylation promotes its nuclear translocation, where it can activate gene expression programmes regulating cell proliferation, differentiation, and migration.

*TAZ* knockdown in HAC15 cells attenuated RSL3‐induced cell death (Figure 7L–N). In low‐density cultures, *TAZ*‐depleted cells displayed enhanced viability compared to controls, while maintaining comparable viability to *TAZ* knockdown cells in high‐density conditions (Figure 7O). Collectively, these findings establish *TAZ* as the principal Hippo pathway effector regulating cell density‐dependent ferroptosis in HAC15 cells, with its activation status being a critical determinant. To extend these findings in APA tissue, we evaluated Hippo signaling activity and *TAZ* expression in spatial transcriptomics‐derived APA cells. Progenitor‐like APA cells exhibited significantly higher Hippo pathway scores compared to more differentiated APA cells (Figure S12A,B, Supporting Information), a trend mirrored by elevated *TAZ* expression in the same cell subset (Figure S12C, Supporting Information). Moreover, both *TAZ* expression (Figure S12D, Supporting Information) and ferroptosis pathway activity (Figure 6K) declined along the pseudotime trajectory toward more mature‐like APA states, suggesting a coordinated regulation. Taken together, these data suggest that *TAZ* may link Hippo signaling to ferroptosis susceptibility in a stage‐specific manner during APA progression.

## 3 Discussion

Our integrated transcriptomic analyses provide novel insights into the cellular and molecular landscapes of APM and APA development, particularly regarding immune cell populations. Myeloid cells, specifically macrophages, emerged as key modulators of the tumor immune microenvironment, with distinct populations present in both APM and APA tissues. A notable shift toward M2‐type macrophages in APA reveals an immunosuppressive microenvironment, a characteristic previously shown to facilitate tumor progression across multiple tumor types.^[^ ^50^, ^51^, ^52^, ^53^, ^54^ ^]^ Our observation of elevated *TREM2* expression in tumor‐infiltrating macrophages extends findings from other tumors, where *TREM2* inhibition has been shown to suppress tumor growth in preclinical studies.^[^ ^55^ ^]^

Cell–cell interaction analyses demonstrated enhanced communication networks in APA compared to APM cells. The neural cell adhesion molecule (NCAM) signaling pathway was significant in APA cells, particularly via NCAM1, but absent in APM cells. These findings suggest that alterations in cell adhesion molecules may function in the pathophysiology of autonomous aldosterone production. This observation partially aligns with the findings of Wu et al., who identified novel CADM1 mutations in APAs, highlighting an unexpected role for cell adhesion molecules and gap junction communication in the regulation of aldosterone production.^[^ ^21^ ^]^

Our analyses revealed a higher likelihood of GAS6‐MERTK mediated information transfer from myeloid cells to APA compared to APM. This observation aligns with previous research demonstrating that GAS6/MERTK signaling induces polarization of M2‐like tumor‐associated macrophages, contributing to the immunosuppressive remodeling of the tumor microenvironment and promoting colorectal cancer progression.^[^ ^37^ ^]^ In contrast, macrophage migration inhibitory factor (MIF)‐related ligand‐receptor interactions were predominantly activated in APMs. This is interesting because MIF has been shown to play a protective role against steatosis and reduce macrophage accumulation,^[^ ^56^ ^]^ suggesting a potential regulatory mechanism in the context of APMs.

Our regulatory network analysis identified key transcription factors, including *LEF1* and *MEIS1*, as APA‐specific regulators, consistent with their roles in adrenocortical cell viability and steroidogenic gene regulation.^[^ ^39^, ^40^ ^]^ Conversely, the prominence of *JUN* and *FOSB* in APM cells aligns with their known roles in early tumor response and adrenal cell proliferation.^[^ ^57^, ^58^ ^]^ The identification of shared transcription factors like ATF4 and ETV3 in APA and APM cells suggests that some regulatory mechanisms persist throughout APA development. Notably, ATF4, which specifically targets the *MCLON1* gene, is involved in stress‐adaptive and calcium‐signaling pathways linked to *KCNJ5* and *ATP1A1* mutations,^[^ ^59^ ^]^ indicating their potential contribution to APA tumorigenesis and suggesting a fundamental regulatory network influencing disease progression.

Previous studies have suggested that aldosterone‐driver mutations may trigger zG remodeling into APMs (also referred to as aldosterone‐producing cell clusters)^[^ ^23^, ^24^, ^27^ ^]^ and that a subset of APMs might progress into APAs,^[^ ^10^, ^60^ ^]^ although molecular evidence in support of this is limited. In this study, we explored tumor initiation and progression integrating analyses of two APA samples using snRNA‐seq with two APM samples from previously published scRNA‐seq data.^[^ ^27^ ^]^ The transcriptomic phenotype of APM was like that of zG cells, positioning them at the developmental starting point, potentially progressing to APA. To delineate zG and APM cells, we utilized spatial transcriptomics in conjunction with CYP11B2 immunohistochemistry, which significantly enhanced our capability to accurately capture APM cells. To delineate the developmental trajectory of APA within an individual patient, we analyzed three captured regions from a single resected adrenal gland containing both APMs and an APA with a *KCNJ5* mutation. This targeted analysis revealed two primary developmental pathways: directly from zG to APA, and from zG through APM to APA. Notably, this dual‐pathway pattern appeared specific to *KCNJ5*‐mutated APAs. In contrast, an integrated spatial analysis of three adrenals with APAs without a *KCNJ5* mutation (comprising an *ATP1A1*‐ and a *CACNA1D*‐mutated APA and an APA with no detected mutation) consistently revealed a single trajectory proceeding from zG to APM and then to APA. These findings suggest that the developmental programs of APA may be shaped by their underlying mutational landscape. Given that *KCNJ5* mutations are rarely detected in APMs,^[^ ^61^ ^]^ our observations support the hypothesis that *KCNJ5*‐mutated APAs can bypass the APM stage and arise directly from zG cells. Supporting this, immunohistochemistry for MDA, a marker of oxidative lipid damage, revealed lower staining levels in *KCNJ5*‐mutated APAs compared to other genotypes. This observation raises the possibility that *KCNJ5* mutations confer a metabolic or redox advantage, allowing these cells to evade oxidative stress checkpoints during adrenal remodeling. This underscores the importance of integrating spatial, transcriptomic, and molecular profiles to resolve the lineage architecture of APA subtypes and to better understand their divergent developmental origins.

We further explored cell differentiation trajectories within APA, identifying two distinct states, progenitor‐like and mature cell states. Cells with higher CytoTRACE scores (progenitor‐like state) demonstrated a less differentiated state with greater developmental potential, suggesting proximity to the developmental origin, which was corroborated by Monocle 2 analysis.^[^ ^62^, ^63^ ^]^ The progenitor‐like state also possessed a higher stemness score, which have been reported as indicator of enhanced differentiation potential.^[^ ^64^ ^]^ Moreover, we employed InferCNV to detect CNV, enabling us to assess the varying tumor characteristics between the two APA states.^[^ ^65^ ^]^ Notably, increased expression of *PCP4* and *CYP11B2* was observed in the mature state with high CNV score. Previous studies have implicated aberrant activation of the WNT signaling pathway in the aetiology of APA,^[^ ^66^ ^]^ and our results further support that this function might be perturbed in early disease stage. Both snRNA‐seq and spatial transcriptomics of APAs highlighted a potential role for ferroptosis in APA pathogenesis.^[^ ^28^, ^31^ ^]^ Indeed, our data indicated an elevated response to oxidative stress in progenitor‐like APA cell state. Furthermore, ferroptosis and lipid peroxidation signalling was found to increase in the development of APA. In the mature APA cell state, ferritin heavy chain 1 (*FTH1*), an iron chelator, was found to be upregulated, aligning with recent findings.^[^ ^28^ ^]^ As the importance and disease relevance of ferroptosis is gaining recognition, various genetic and non‐genetic determinants of ferroptosis are being extensively explored.^[^ ^67^ ^]^ Among non‐genetic factors, cell density and cell–cell interactions often modulate intercellular communications and mechanical forces to affect ferroptosis resistance.^[^ ^49^, ^67^ ^]^

Our in vitro experiments and RNA‐seq analysis revealed a relationship between cell density and ferroptosis sensitivity in human adrenocortical cells. Of note, we identified the Hippo pathway as a key regulator in this process, with *TAZ* activation potentially serving as a promoter and predictor of ferroptosis sensitivity, a phenomenon also observed in other cell lines, such as renal cell carcinoma^[^ ^49^ ^]^ and epithelial ovarian cancer.^[^ ^68^ ^]^ These findings expand our understanding of non‐genetic factors that influence adrenal cell ferroptosis and suggest that cellular context, particularly cell density, plays a key role in determining ferroptosis outcomes.

We report a comprehensive, integrative analysis of aldosterone‑producing lesions using both single‑cell/nucleus and spatial transcriptomics, offering high‑resolution insights into cellular heterogeneity, gene regulatory networks, and developmental trajectories associated with *KCNJ5* mutations. The combination of spatial and single‑cell modalities, together with functional validation experiments, enables robust biological interpretation and reveals novel mechanisms such as ferroptosis adaptation and cell density‑dependent signaling. Nevertheless, several limitations should be acknowledged. First, limited representation of certain genotypes hampers validation of the developmental trajectory findings and ion channel‐specific mechanisms; future studies should include larger, genotype‑stratified cohorts with balanced representation of both sexes to enable more definitive conclusions on genotype‐driven developmental pathways and determine how different forms of ionic dysregulation shape APA ontogeny. Second, genotype data for APMs were unavailable in the public scRNA‑seq dataset owing to missing mutation metadata and could not be obtained from our spatial transcriptomics samples because the small size of APMs and tissue depletion after repeated sectioning prevented sufficient DNA extraction.

In conclusion, our integrated multi‐omics analyses reveal the complex molecular landscape of APM and APA progression, characterized by distinct regulatory networks and an immunosuppressive microenvironment marked by M2‐macrophage enrichment. The identification of developmental trajectories between APM and APA cells, alongside the characterization of cell–cell communication networks, provides a molecular framework for understanding primary aldosteronism pathogenesis, whilst highlighting the dynamic cellular interactions that influence disease progression.

## 4 Experimental Section

### Patients and Sample Collection

This research was conducted following the guidelines of the local ethics committee and was approved by the institutional review board at the Ludwig‐Maximilian University of Munich (ref. 379‐10 and 24–0696). All samples were collected at the University Hospital, and informed consent was obtained from all patients. Surgically removed adrenals from two male patients diagnosed with unilateral PA and with a histopathological classification of an APA were used for snRNA‐seq.^[^ ^69^ ^]^ To address the limited snRNA‐seq sample size of APA, we incorporated two publicly available snRNA‐seq or scRNA‐seq datasets comprising 11 *KCNJ5*‐mutated APA samples (syn6023883, n = 8 and GSE242404, n = 3).^[^ ^28^, ^30^ ^]^ In addition, data from five peritumoral adrenal tissues adjacent to APAs (*KCNJ5*‐mutated APA, n = 2 and *ATP2B3*‐mutated APA, n = 3) from GSE210381 were used to evaluate the evolutionary trajectory of adrenal tissues.^[^ ^41^ ^]^ The E‐MTAB‐11837 dataset was downloaded from the European Bioinformatics Institute (EBI, https://www.ebi.ac.uk/), including 2 APM tissues obtained from 2 male patients.^[^ ^27^ ^]^ Twelve FFPE specimens derived from 6 patients, containing 3 APA and 17 APM areas, were used for ST analysis.

### Hematoxylin and Eosin Staining and Immunohistochemistry of Adrenal Samples

4 µm thick serial sections of the FFPE tissues were prepared for H&E and IHC staining as previously described.^[^ ^70^, ^71^ ^]^ Briefly, tissue sections were de‐paraffinized in xylene and rehydrated with graded ethanol and used for H&E staining and immunohistochemistyry. For the latter, the slides were blocked and incubated with antibodies against CYP11B2 (1:200, clone 17B),^[^ ^72^ ^]^ CD206 (1:400, Cell Signaling Technology #24595), or MDA (1:200, JaICA, MMD‐030, clone 1E83) overnight at 4 °C. The tissue sections were observed under the uSCOPE MXII digital microscope.

### Whole Exome Sequencing of APA Tissue Samples

Sample specimens from two APAs from different patients in our cohort were dissected, snap‐frozen and pulverized in liquid nitrogen. Genomic DNA was extracted (DNeasy Blood & Tissue Kit, QIAGEN, 69504) for whole exome sequencing (WES). Sequencing using 150‐bp paired‐end reads was performed on a DNBseq platform (BGI Global Genomic Services, Hong Kong, China) with 0.5 µg of genomic DNA per sample. Sequencing‐derived raw image files were processed using DNBseq base‐calling software with default parameters. The clean reads were aligned to the human assembly hg38 (hg38/GRCh38) with the Burrows‐Wheeler Aligner tool (BWA‐MEM, v0.7.17).^[^ ^73^ ^]^ BAM files were created using Picard tools followed by base quality score recalibration using known indels and single nucleotide polymorphism (SNP) from the 1000 Genomes Project with GATK (v4.1.4.1).^[^ ^74^ ^]^ Somatic mutations were called from the WES data using MuTect2^[^ ^75^ ^]^ in GATK, incorporating a panel of normals (PoN) and polymorphic site data from gnomAD.^[^ ^76^ ^]^ The resulting raw variant call format (VCF) files were filtered with the FilterMutectCalls module and functionally annotated using Funcotator from GATK.

### KCNJ5 Genotyping

Immunohistochemistry of CYP11B2 adrenal sections was used to guide macrodissection of regions corresponding to the CYP11B2‐positive tumor region from subsequent unstained sections.^[^ ^77^ ^]^ The QIAamp DNA FFPE Tissue Kit (QIAGEN, 56 404) was used to isolate genomic DNA that was then used for sequencing *KCNJ5* and mutation analysis.

### Adrenal Tissue Processing and Single Nuclei Isolation

Nuclei isolation of cryofrozen APA tissue samples was performed using the Chromium Nuclei Isolation Kit with RNase Inhibitor (10x Genomics, PN‐1000494) according to the manufacturer's protocol. Briefly, small pieces (≈1 mm^3^) of adenoma were dissected from cryofrozen adrenal tissue samples, homogenized in lysis buffer, and incubated on ice for 10 min. The dissociated tissue was transferred onto a column and centrifuged at 500 *g *for 3 min at 4 °C. The pellet was washed twice with 1 mL wash buffer and the isolated nuclei were resuspended in resuspension buffer, intact nuclei was confirmed microscopically, and counted using Countess 3 (Invitrogen, AMQAX2000).

### Single‐Nuclei Library Construction and Sequencing

Library preparation was generated as described in the User's Guide for Chromium Next GEM Single Cell Multiome ATAC + Gene Expression Kit (10x Genomics, PN‐1000285). Library quality control was performed using the 2100 Bioanalyzer (Agilent Technologies) prior to sequencing. All libraries were sequenced on the Illumina NextSeq 2000 platform to generate 150‐bp paired‐end reads.

### snRNA‐seq Data Preprocessing and Quality Control

FASTQs were inputted into the Cell Ranger ARC pipeline (v2.0.2, 10x Genomics) to generate barcoded count matrices of gene expression data for each sample with the “cellranger‐arc count” command and default parameters. Raw sequencing reads were mapped to the human genome assembly GRCh38. Downstream analyses were performed using Seurat R package (v5.1.0).^[^ ^78^ ^]^ For quality control, genes with counts in fewer than 3 nuclei were filtered out to exclude genes likely detected due to random noise. Low‐quality nuclei were removed using the unique molecular identifier (UMI) counts (nCount_RNA > 300), genes (500 < nFeature_RNA < 7500), and the proportion of mitochondrial genes (percent.mito < 20). The DoubletFinder (v2.0) R package was also utilized to remove potential doublets with the default settings.^[^ ^79^ ^]^

### Downstream Clustering and Cell Annotation

Data were normalized using the *NormalizeData* function with default parameters to remove differences in sequencing depth across cells. We then used the standard Seurat clustering pipeline with the following functions: *FindVariableFeatures* function was used to find the top 2000 highly variable genes, *ScaleData* function was used to scale and centre these genes in the integrated matrices, and then scaled data were subjected to linear dimensional reduction through principal component analysis (PCA) with the *RunPCA* function. After comparing the five established batch correction methods (Canonical Correlation Analysis, Reciprocal Principal Component Analysis, Harmony, Fast Mutual Nearest Neighbors, and single‐cell Variational Inference), Harmony was selected as the best batch correction approach for data integration. Subsequently, *FindNeighbors* and *FindClusters* functions were used to cluster the first 20 principal components based on the knee point of the scree plot and project them to 2D UMAP images for display. The expression of each gene in each cluster was compared against the rest of the clusters using the Wilcoxon rank‐sum test with the *FindAllMarkers* function. The significantly differentially expressed genes (DEG) with an adjusted *P* value < 0.05 were retained for further analysis. The corresponding cell types of the clusters were annotated manually following the expression of known marker genes of the respective clustering results.

### Estimation of Differential Cell Density

Differential cell density was estimated using the *estimateCellDensity* function from Cacoa^[^ ^80^ ^]^ as in previous studies.^[^ ^81^, ^82^ ^]^ Initially, the kernel density within the joint embedding space was computed for each sample using the ks R package (bin = 400) to quantitatively assess differential cell density between APA and APM groups. To eliminate systemic biases across samples, we then performed quantile normalization on the obtained density matrix. To precisely impute the differential cell density between sample groups, we conducted *t*‐tests between sample groups in each gridded bin. To minimize background noise interference, only bins containing at least one cell were considered. The resulting *Z* scores were illustrated using heatmaps.

### Compositional Data Analysis

To mitigate the potential skew from diverse cell clusters, we utilized the *estimateCellLoadings* function from Cacoa^[^ ^80^ ^]^ for compositional data analysis (CDA), as described in prior studies,^[^ ^81^, ^83^ ^]^ to compare robust estimates of compositional changes. In brief, this analysis employed isometric log‐ratio transformations on cell type fractions, which were subsequently analysed using canonical discriminant analysis via the candisc R package. The calculated separating coefficients from these iterations were plotted to visually depict compositional differences.

### Cell Communication Analysis using CellChat

To assess cellular crosstalk between different cell types, intercellular interactions based on ligands and receptors were inferred and visualized by CellChat.^[^ ^36^ ^]^ In brief, gene expression data of cells and assigned cell type were used as input and core functions were run with standard parameters to infer communication networks and signaling pathways. Interaction events were categorized into secretory signals, cell–cell contact, and extracellular matrix (ECM) receptors based on the molecular characteristics of the ligand‐receptor pair. Special attention was given to the dynamics of interaction changes, such as the gain or loss of interactions among general cell types and the shifts in outgoing and incoming interactions within cell clusters.

### Molecular Docking Analysis

Molecular docking of receptor‐ligand pairs was implemented using Autodock Vina (v1.2.x) to identify appropriate binding modes.^[^ ^84^ ^]^ The protein crystal structure of *GAS6* and *MERTK* was available in the Protein Database^[^ ^85^ ^]^ and used for molecular modeling studies. Binding energy < 0 was taken to indicate possible binding. The best binding conformation was selected with the lowest binding energy and further visualization was performed using PyMol.^[^ ^86^ ^]^

### Gene Regulatory Network Analysis

To identify cell type‐specific gene regulatory network and regulon activity, single‐cell regulatory network inference and clustering (SCENIC) analysis were performed using pySCENIC (v0.12.1).^[^ ^87^ ^]^ In short, default parameters were used for the SCENIC workflow, and the raw count matrix from all samples was used as input. The co‐expression network was calculated by *runGenie3* and regulons were identified by *RcisTarget*. Finally, the activity of each regulon in each cell was evaluated using the AUCell algorithm. Further insights into regulon specificity for each cell type were obtained through regulon specificity scores (RSS). Hierarchical clustering using Euclidean distance was conducted based on the connection specificity index (CSI) to identify distinct regulon modules.^[^ ^88^, ^89^ ^]^

### Gene Ontology and Pathway Activity Scoring

Gene ontology and pathway analyses for DEGs in each cluster were performed using the clusterProfiler R package. The expression activity of a given gene set/pathway was quantified using the *AddModuleScore* function in Seurat or the AUCell algorithm. The precompiled canonical pathway gene sets in the molecular signatures database (MSigDB) were applied in this analysis. Some known functional gene sets of macrophages are displayed in Table S11 (Supporting Information). Enrichments were regarded as statistically significant with an adjusted *P* value < 0.05.

### Spatial Transcriptomics Sample Preparation, Library Construction, and Sequencing

10x Visium spatial gene expression analysis was performed on FFPE adrenal tissue sections derived from patients with PA. Total RNA was extracted from all FFPE sections using the RNeasy FFPE Kit (QIAGEN, 73504) for quality control with the 2100 Bioanalyzer (Agilent Technologies). The FFPE sections mounted on capture areas (6.5 × 6.5 mm) of spatial gene expression slides (Visium, 10x Genomics, PN‐1000185) were used for H&E staining and bright field imaging (Zeiss Axio Imager 2) before tissue permeabilization and cDNA library preparation (Visium, 10x Genomics). Post‐library construction quality was evaluated with the 2100 Bioanalyzer (Agilent Technologies). Samples were sequenced with the Illumina NextSeq 2000. The paired end, dual indexing read protocol included the following cycle numbers: Read 1, 28 cycles; i5 and i7 indexes, 10 cycles; Read 2, 90 cycles.

### Processing of the Spatial Transcriptomic Data

Demultiplexed FASTQs were obtained using je‐suite.^[^ ^90^ ^]^ Using the Visium Manual Alignment Wizard, the image was aligned with the fiducial frames on the Visium slide before the tissue containing capture spots were selected manually for further analyses. Based on this, sequencing data were aligned to the human reference genome (GRCh38) using the Space Ranger pipeline (v1.3.1, 10x Genomics). Seurat was used to derive a feature spot‐barcode expression matrix with an H&E‐stained brightfield image and FASTQ files as inputs. Gene expression data were normalized by the *SCTransform* function in Seurat, which uses regularized negative binomial models to suppress technical artifacts while preserving biological variance. To eliminate the batch effect, different spatial transcriptomics samples were integrated through the default parameters of the *FindIntegrationAnchors* and *IntegrateData* functions. Integrated ST data were processed similarly to the single cell and single nuclei data, involving dimensionality reduction with the *RunPCA* function, clustering of ST spots using *FindNeighbors* and *FindClusters* functions, and visualization with *RunUMAP* function.

### Pseudotime Analysis using Monocle2

Pseudotime trajectories for the main cell types were constructed using Monocle 2 to reveal the dynamic process of cell and gene expression changes.^[^ ^63^ ^]^ RNA counts from the interest of cell types were selected and genes expressed in less than 10 cells were retained for downstream analysis. The ordering genes along pseudotime were determined using *differentialGeneTest* function with an adjusted *P* less than 0.01. Dimensionality reduction and trajectory construction were then performed with default parameters through the *reduceDimension* function with DDRTree method. Additionally, cells were color‐labeled according to the cell type identity classified by Seurat to ensure meaningful ordering. Finally, the *plot_cell_trajectory* and *plot_pseudotime_heatmap* functions were used for visualization.

### Cell Differentiation Root Inference using CytoTRACE

The CytoTRACE algorithm was employed with default parameters to evaluate cellular differentiation states by analyzing gene expression profiles.^[^ ^62^ ^]^ Genes were ranked according to the correlation between their expression levels and the number of expressed genes per spot. The expression of the highest correlating genes was then aggregated to compute the CytoTRACE score. CytoTRACE scores range from 0 to 1, while higher scores indicate higher stemness (less differentiation) and vice versa.^[^ ^91^ ^]^

### Spatial Inferred Copy Number Variation (inferCNV) Analysis

The assessment of CNV within individual spots was meticulously executed utilising the inferCNV algorithm,^[^ ^65^ ^]^ with a specific focus on the CNVs of APA cell subtypes. The data analysis was performed by calculating the transcription intensity of genes across various locations on the genome of APA cells from each spot in comparison with a set of reference spots (zG cells). The modified CNV level (relative CNV expression) of genes on each chromosome was illustrated in a heatmap. The distribution of CNV scores in different APA states was compared to identify the degree of cellular alteration.

### Cell Lines and Cell Culture

Human adrenocortical (HAC15) cells were cultured at 37 °C and 5% CO_2_ in Dulbecco's Modified Eagle's Medium/Nutrient Mixture F‐12 ((DMEM/F12, Gibco) supplemented with 10% Cosmic Calf Serum (CCS, Hyclone), 1% insulin‐transferrin‐selenium (ITS, Gibco), 1% antibiotic‐antimicotic (Gibco), and 0.01% Gentamycin (Gibco). Unless otherwise stated, HAC15 cells were cultured for 24‐h and then incubated for a further 24‐h in starvation medium using DMEM/F12 containing 0.1% CCS before experimental treatments.

### Bulk RNA Sequencing and Pathway Analysis

HAC15 cells^[^ ^92^ ^]^ were cultured in 6‐well plates at different densities (low‐density: 0.5 × 10^6^cell/well and high‐density: 2.0 × 10^6^ cell/well) and subsequently treated with 4 µM[1S, 3R]‐RSL3 (RSL3, TOCRIS, 6118) for 3 h in biological triplicates. After that, total RNA was extracted using a Maxwell simplyRNA Cells Kit (Promega, AS1390) and then a sequencing library was prepared according to the manufacturer's instructions. mRNA sequencing was conducted by Eurofins Genomics (Konstanz, Germany) using Illumina paired‐end sequencing with a read length of 150‐bp on the NovaSeq 6000 platform. Differential expression was analysed using the DESeq2 R package, and significant genes were filtered by an adjusted *P* value < 0.05, and |log(fold change)| > 2. The clusterProfiler R package was used to find different enriched GO terms between distinct groups. An adjusted *P* value < 0.05 was used to determine significant gene set enrichment.

### Generation of Adrenal Cells with TAZ‐Specific Knockdown

HAC15 cells were transfected with 2 µL of a 100 µM TAZ siRNA (Dharmacon, M‐016083‐00‐0002) using the Cell Line Nucleofector Kit R (Amaxa, VCA‐1001) and the Nucleofector 2b device (Lonza, program X‐005) according to the manufacturer's instructions. Transfected cells were harvested for real‐time qPCR analysis after 48‐h or Western blot analysis after 72‐h to measure levels of gene or protein expression, respectively.

### Flow Cytometry

HAC15 cells cultured at different cell densities were seeded into 6‐well plates and incubated for 24‐h, followed by a further 24‐h in starvation medium. Cells were then treated with 3 µM RSL3 for 3‐h. Subsequently, all cells were harvested and stained with propidium iodide (PI, eBioscience, 00‐6990‐50) to detect cell death. Fluorescence intensity was measured using an LSRFortessa cell analyzer (BD Bioscience, 647794) on the PE‐Texas Red channel. Data analysis was conducted using FlowJo software (version 10.8.1).

### Cell Viability Assay

The effect of RSL3 on HAC15 cell viability was assessed using the water‐soluble tetrazolium salt‐1 assay (WST‐1, Roche, 11644807001). Briefly, HAC15 cells were plated in starvation medium into 96‐well plates and incubated for 24‐h before treatment with RSL3 or 10 µM Lip‐1 (TOCRIS, 6113) + 4 µM RSL3 for 4‐h. After incubation with WST‐1 solution at 37 °C and 5% CO_2_ for 3‐h, absorbance at 450 nm and 690 nm were determined on a FLUOstar Omega plate reader (BMG LABTECH).

### Real‐Time qPCR Analysis

Total RNA was extracted from cells using Maxwell RSC simplyRNA Cells Kit (Promega, AS1390) and reverse transcription was performed on 500 ng RNA (GoScript reverse transcriptase kit, Promega, A2791) according to the manufacturers’ instructions. Universal Probes Supermix (Bio‐rad, 1725131) was used to perform real‐time qPCR on the QuantStudio 5 System (Thermo Fisher Scientific) using the TaqMan probe for TAZ (Hs00210007_m1). GAPDH (Hs02786624_g1) was used for normalization, and the 2^−∆∆Ct^ relative quantification method was used for calculating gene expression levels.

### Western Blot Analysis

Western blotting was performed as previously described.^[^ ^93^ ^]^ The cytoplasmic and nuclear protein extracts were prepared using the Nuclear and Cytoplasmic Extraction Reagents (Thermo Fisher Scientific, 78835). The primary antibodies used in this study were listed in Table S12 (Supporting Information). Images were acquired on ChemiDoc XRS+ System (Bio‐Rad).

### Immunofluorescence

Immunofluorescence staining was conducted as reported previously.^[^ ^71^ ^]^ Cells were incubated overnight at 4 °C with SYTOX green (1:500, Cell Signaling Technology, 4153), or antibodies against TfR1 (1:500, Sigma‐Aldrich, MABC1765), or TAZ (1:200, Cell Signaling Technology, 83669). Images were captured on a Leica DM2500 microscope, and three microscopic fields of each sample were randomly selected.

### Statistical Analysis

Statistical analyses were performed using R (version 4.3.2) and GraphPad Prism 9. For comparisons of continuous variables between two groups, either the Student's *t*‐test or the Wilcoxon rank‐sum test was applied. Two‐way ANOVA was applied for analyses involving two independent factors, with Tukey's post hoc test used for multiple comparisons when appropriate. Categorical data were analyzed using Fisher's exact test. All experimental results are presented as mean ± SD unless otherwise specified. A two‐sided *P* value < 0.05 was considered statistically significant.

## Conflict of Interest

The authors declare no conflict of interest.

## Author Contributions

Z.S. and T.A.W. conceived the study and formulated research objectives and aims; Z.S., C.J., M.T., and S.G. conducted transcriptomics data acquisition; Z.S. and C.J. performed bioinformatics analysis and data interpretation; Z.S., M.T., and S.G. conducted in vitro experiments; M.T., S.G., J.W., and Y.P. collected and processed samples and data; Z.S. and T.A.W. wrote the original draft of the manuscript. T.A.W. supervised the research; T.A.W. and M.R. acquired funding for the work; M.R. provided laboratory resources and contributed critical discussions. All authors reviewed and edited the manuscript and approved the final version for submission.

## Supporting information



Supporting Information

Supplemental Table

## Data Availability

The data that support the findings of this study are available from the corresponding author upon reasonable request.
