# Supplementary material for: Integrated Transcriptomics Reveals Evolutionary Trajectories and Cell Density‐Dependent Mechanisms in Aldosterone‐Producing Adenomas
Source: Adv Sci (Weinh). 2026 Jan 21;13(32):e05410. doi: 10.1002/advs.202505410 (PMC13252614; doi:10.1002/advs.202505410)
Supplement: Supplementary file 1 — Supporting Information [file ADVS-13-e05410-s001.docx]

Supporting Information

**Integrated Transcriptomics Reveals Evolutionary Trajectories and Cell Density–Dependent Mechanisms in Aldosterone-Producing Adenomas**

*Zhuolun Sun, Changying Jing, Martina Tetti, Siyuan Gong, Jia Wei, Yingxian Pang, Martin Reincke, Tracy Ann Williams**

Medizinische Klinik und Poliklinik IV, Klinikum der Universität München, Ludwig-Maximilians-Universität München, Munich 80336, Germany (Z. Sun, M. Tetti, S. Gong, J. Wei, Y. Pang, M. Reincke, T.A. Williams).

Institute of Diabetes and Regeneration Research, Helmholtz Munich, Neuherberg, Germany; German Center for Diabetes Research (DZD), Neuherberg, Germany; Munich Medical Research School, Ludwig-Maximilians-Universität, Munich 80539, Germany (C. Jing).

*Corresponding author; E-mail: [Tracy.Williams@med.uni-muenchen.de](mailto:Tracy.Williams@med.uni-muenchen.de)

**Contents**

**Supplementary Figures­**

Figure S1. Relative abundance of cell types… page 2

Figure S2. Enrichment of immunosuppressive macrophages in APA … page 3

Figure S3. Captured adrenal regions for spatial transcriptomics… page 4

Figure S4. Spatial cluster distribution (left) and CYP11B2 gene expression (right) of 12 adrenal sections… page 5

Figure S5. CD206 immunohistochemistry of aldosterone-producing adenomas according to genotype… page 6

Figure S6. Cell-cell contact phenotype and preferential signaling pathways in APA cells… page 7

Figure S7. Pseudotime trajectory analysis of zG, APM, and APA adrenal cell populations at the single-cell and single-nucleus level… page 8

Figure S8. Pseudotime trajectory analysis of zG, APM, and APA cells at the spatial level in *KCNJ5*-mutated APAs… page 9

Figure S9. Pseudotime trajectory analysis of zG, APM, and APA cells at the spatial level in APAs without a *KCNJ5* mutation… page 10

Figure S10. Dynamic changes of biological processes and gene expression during APA progression… page 11

Figure S11. Malondialdehyde immunohistochemistry of aldosterone-producing adenomas according to genotype… page 12

Figure S12. Stage-specific Hippo signaling and *TAZ* expression dynamics along the APA pseudotime trajectory in spatial transcriptomic data… page 13

**
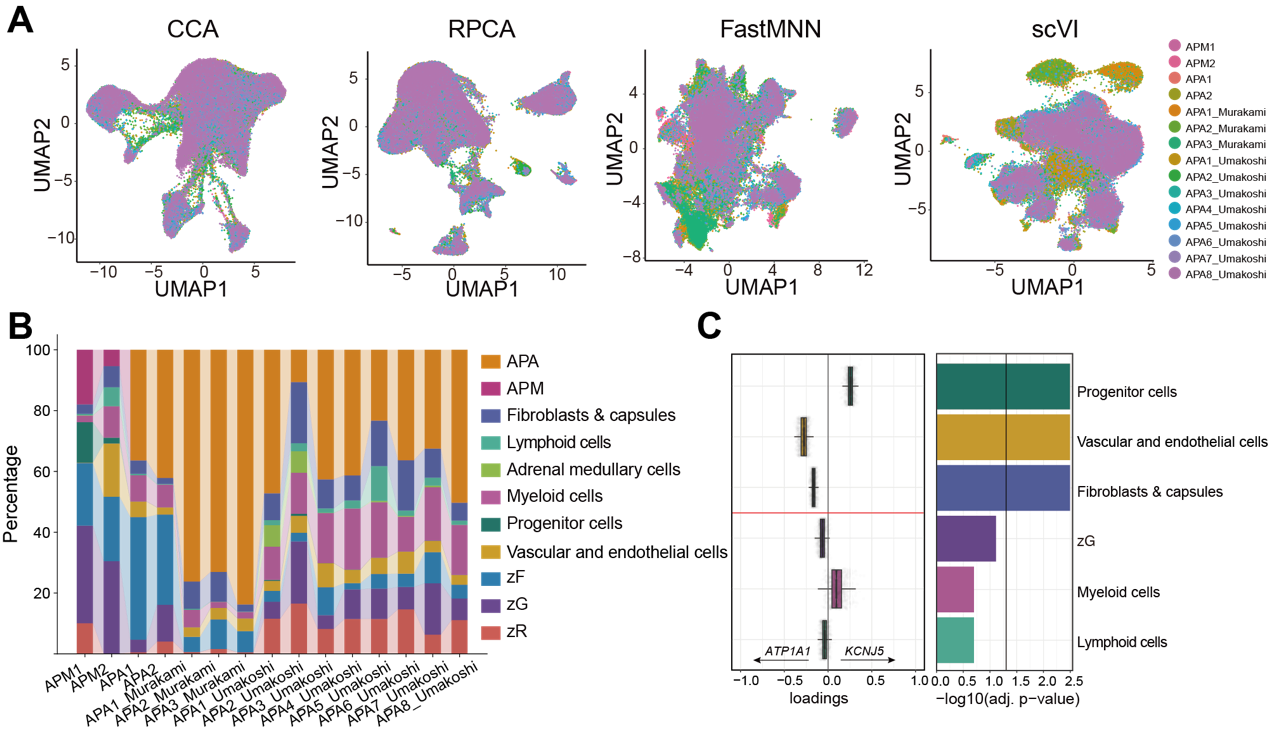
**

**Figure S1**. **Relative abundance of cell types.** Related to Figure 1 in the main manuscript: A) The effect of four different integrative methods for the analysis of cell types in 13 APA and 2 APM samples, visualized using a common UMAP embedding. B) Stacked bar chart showing the relative abundance of each cell type across the 15 samples. C) Changes in cell composition were assessed using Compositional Data Analysis. The x-axis represents the separation coefficient for each cell type, where positive values indicate increased abundance and negative values signify decreased abundance in the *KCNJ5*-mutated APA. Boxplots and individual data points depict uncertainty derived from bootstrap resampling of samples and cells. APA, aldosterone-producing adenoma; APM, aldosterone-producing micronodule; CCA, canonical correlation analysis; FastMNN, fast mutual nearest neighbors; RPCA, reciprocal principal component analysis; scVI, single-cell variational inference; UMAP, uniform manifold approximation and projection; zF, zona fasciculata; zG, zona glomerulosa; zR, zona reticularis. **
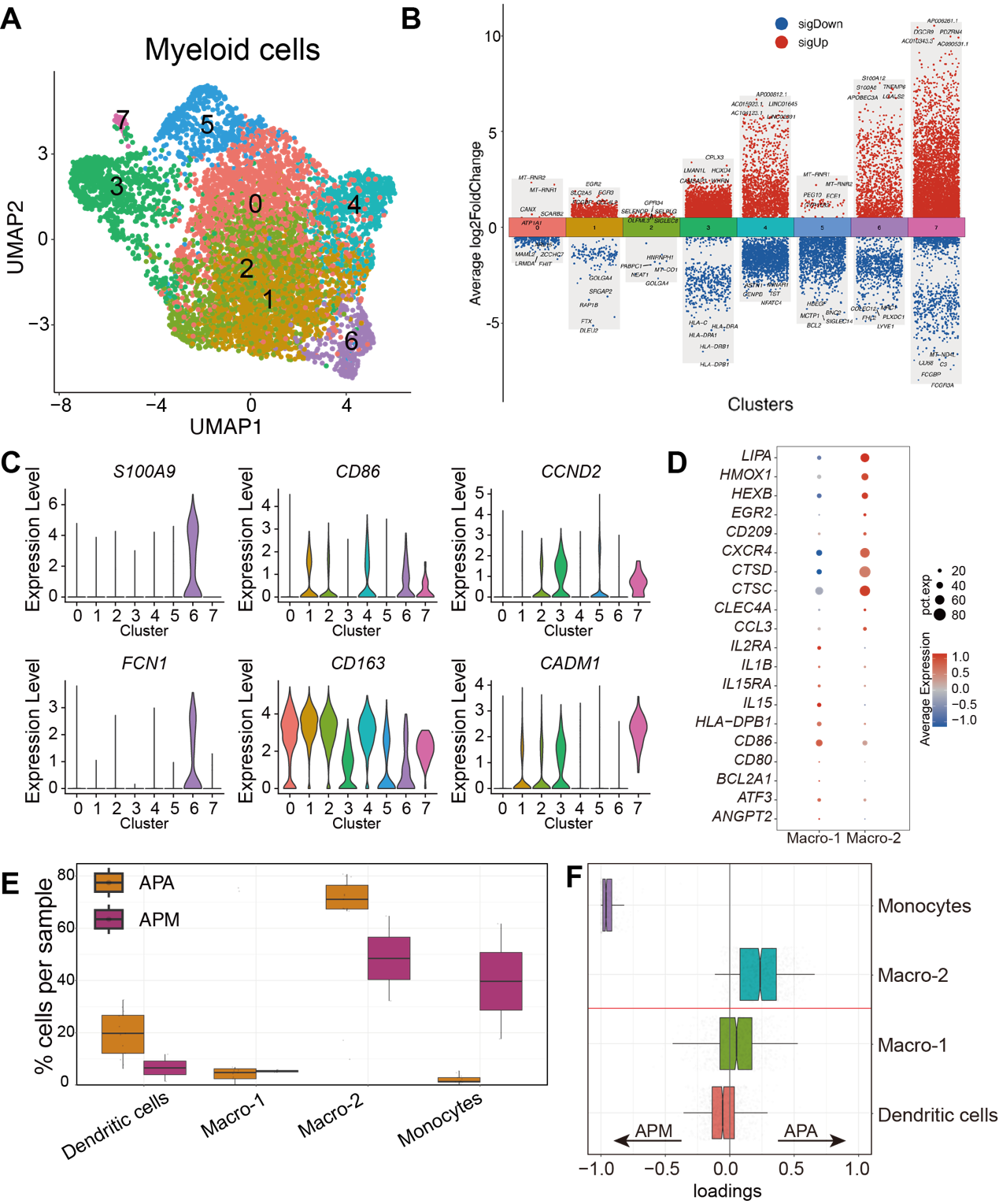
Figure S2. Enrichment of immunosuppressive macrophages in APA.** Related to Figure 2 in the main manuscript: A) UMAP plot of eight myeloid cell clusters: 0 (red), 1 (goldenrod), and 2 (lime) for macrophage-2; 4 (cyan) for macrophage-1; 3 (green), 5 (blue), and 7 (magenta) for dendritic cells; 6 (violet) for monocytes. B) Differentially expressed genes in the eight major cell clusters (one vs. others, Wilcoxon test, |log2FC| > 0.5, adjusted P < 0.05). C) Violin plot of cell type-specific markers: *S100A9, FCN1* (monocytes); *CD86* (macrophage-1); *CD163* (macrophage-2); *CCND2, CADM1* (dendritic cells). D) Bubble plot of marker gene expression in macrophage populations: color shows scaled average expression; size indicates the proportion of cells expressing each gene. E) Changes in the composition of the myeloid compartments between APA and APM. F) Changes in cell composition were assessed by compositional data analysis. The x‑axis shows the separation coefficient per cell type (positive = increased, negative = decreased abundance in APA). Boxplots and points indicate uncertainty from bootstrap resampling.

**
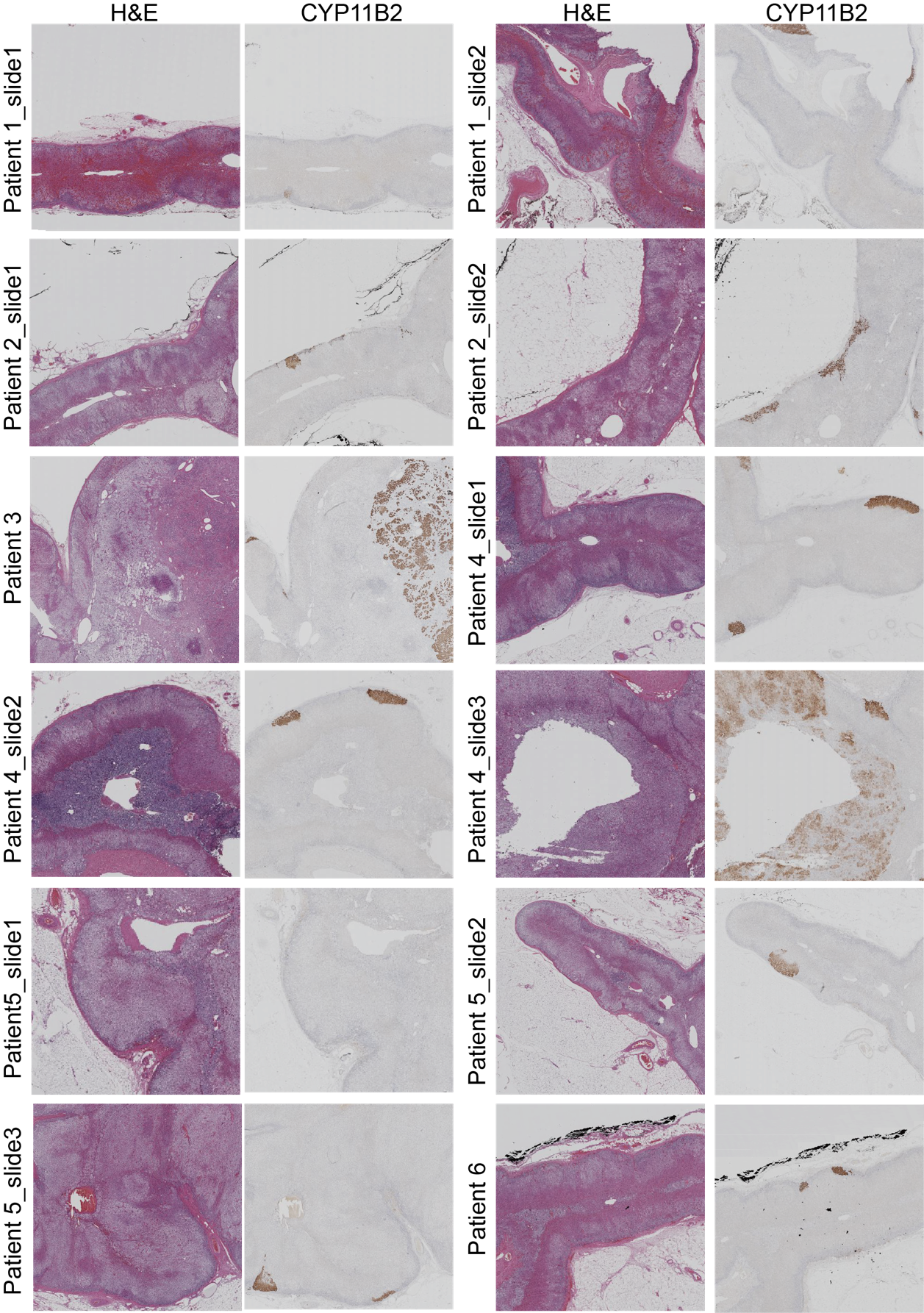
Figure S3. Captured adrenal regions for spatial transcriptomics.** Related to Figure 2 in the main manuscript: Formalin-fixed paraffin-embedded sections used for spatial transcriptomics (10X Genomics Visium platform) with H&E (left) and CYP11B2 IHC (right) staining. Patient 5, slide 1 was selected to ensure that at least one sample included in spatial transcriptomic profiling represented histologically normal adrenal adjacent tissue, without evidence of APM or APA. H&E, hematoxylin and eosin; IHC, immunohistochemistry.


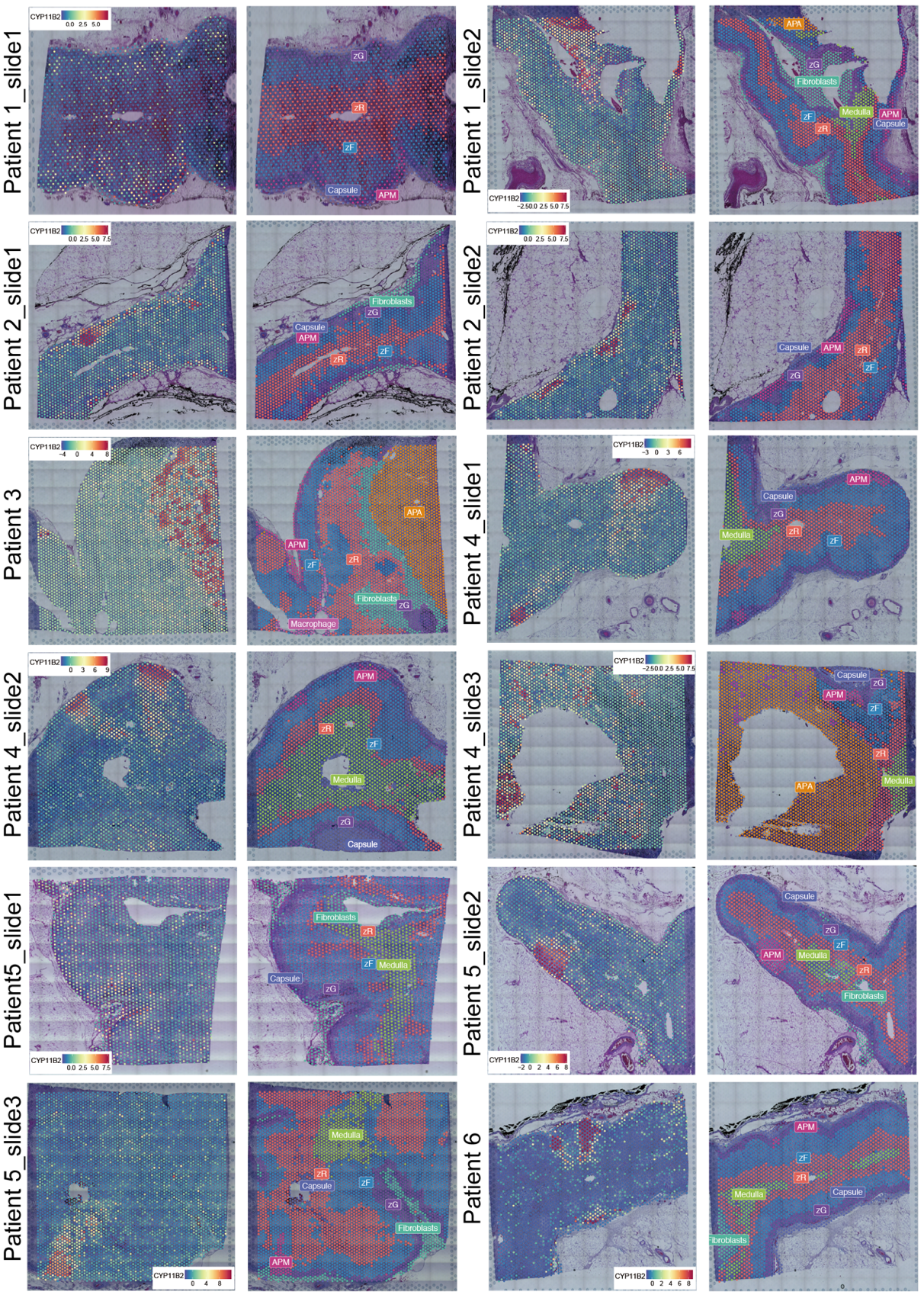
**Figure S4. Spatial cluster distribution (left) and *CYP11B2* gene expression (right) of 12 adrenal sections.** Related to Figure 2 in the main manuscript: Gene expression data were normalized using the SCTransform function in Seurat. Each individual ST sample underwent processing, including dimensionality reduction with the RunPCA function, spot clustering using the FindNeighbors and FindClusters functions, and visualization with the RunUMAP function. APA, aldosterone-producing adenoma; APM, aldosterone-producing micronodule; ST, spatial transcriptomics; zF, zona fasciculata; zG, zona glomerulosa; zR, zona reticularis.


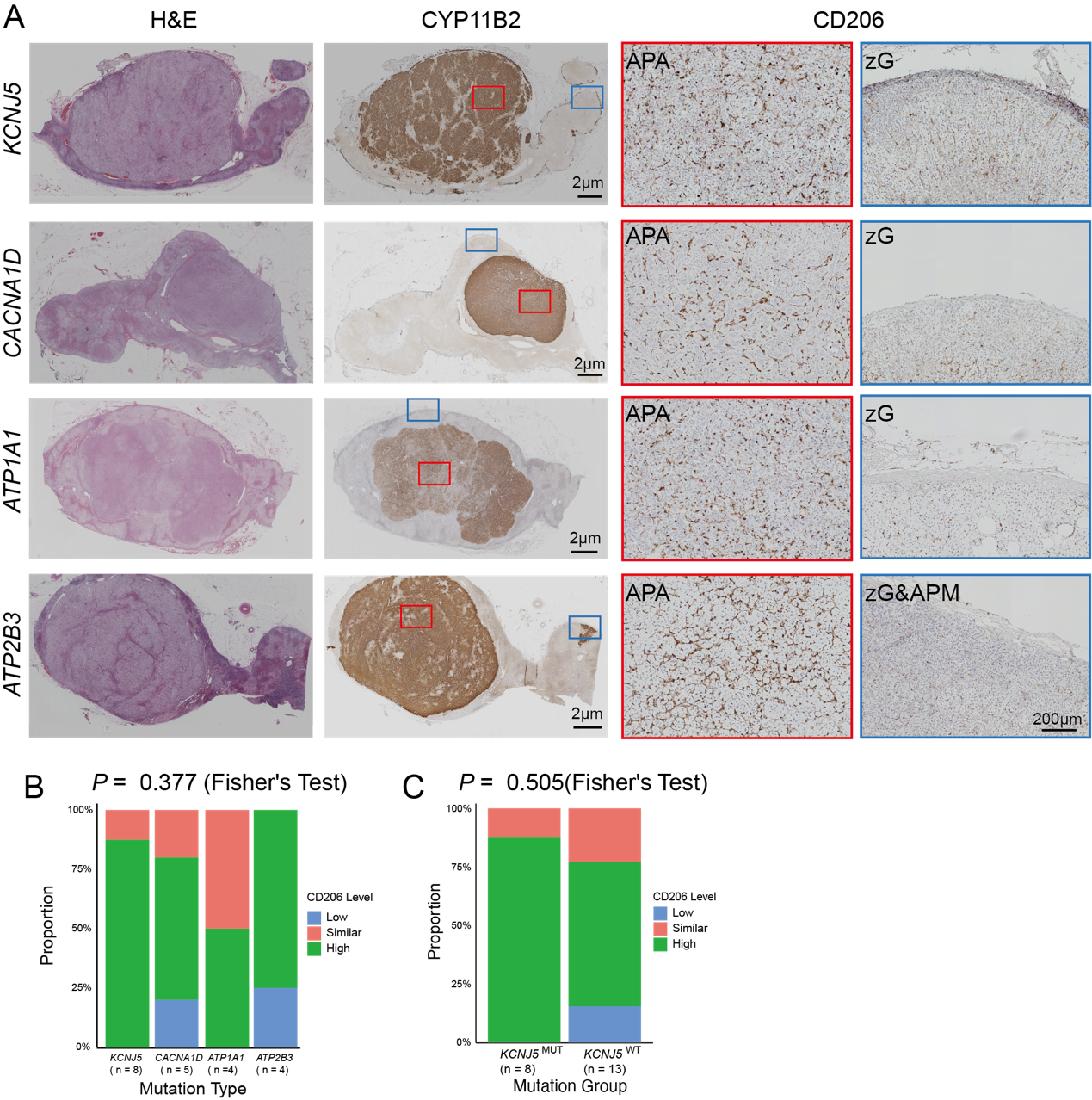


**Figure S5. CD206 immunohistochemistry of aldosterone-producing adenomas according to genotype**. Related to Figure 2 in the main manuscript: A) The H&E staining and immunohistochemistry of CYP11B2 and CD206 in adrenal sections with an APA with mutations in either *KCNJ5* (n = 8), *CACNA1D* (n = 5), *ATP1A1* (n = 4) or *ATP2B3* (n = 4) or as indicated. A representative image is shown for each mutation type. B) Proportion of CD206 staining levels (High, Similar, Low) across APA samples harboring mutations in *KCNJ5*, *CACNA1D, ATP1A1* or *ATP2B3*. Statistical comparison was performed using Fisher’s exact test (P = 0.377). C) CD206 staining levels in female (n = 4) and male (n = 4) patients within the *KCNJ5*-mutated APA group. Statistical comparison was performed using Fisher’s exact test (*P* = 0.553). APA, aldosterone-producing adenoma; APM, aldosterone-producing micronodule; H&E, hematoxylin and eosin; zG, zona glomerulosa.

**
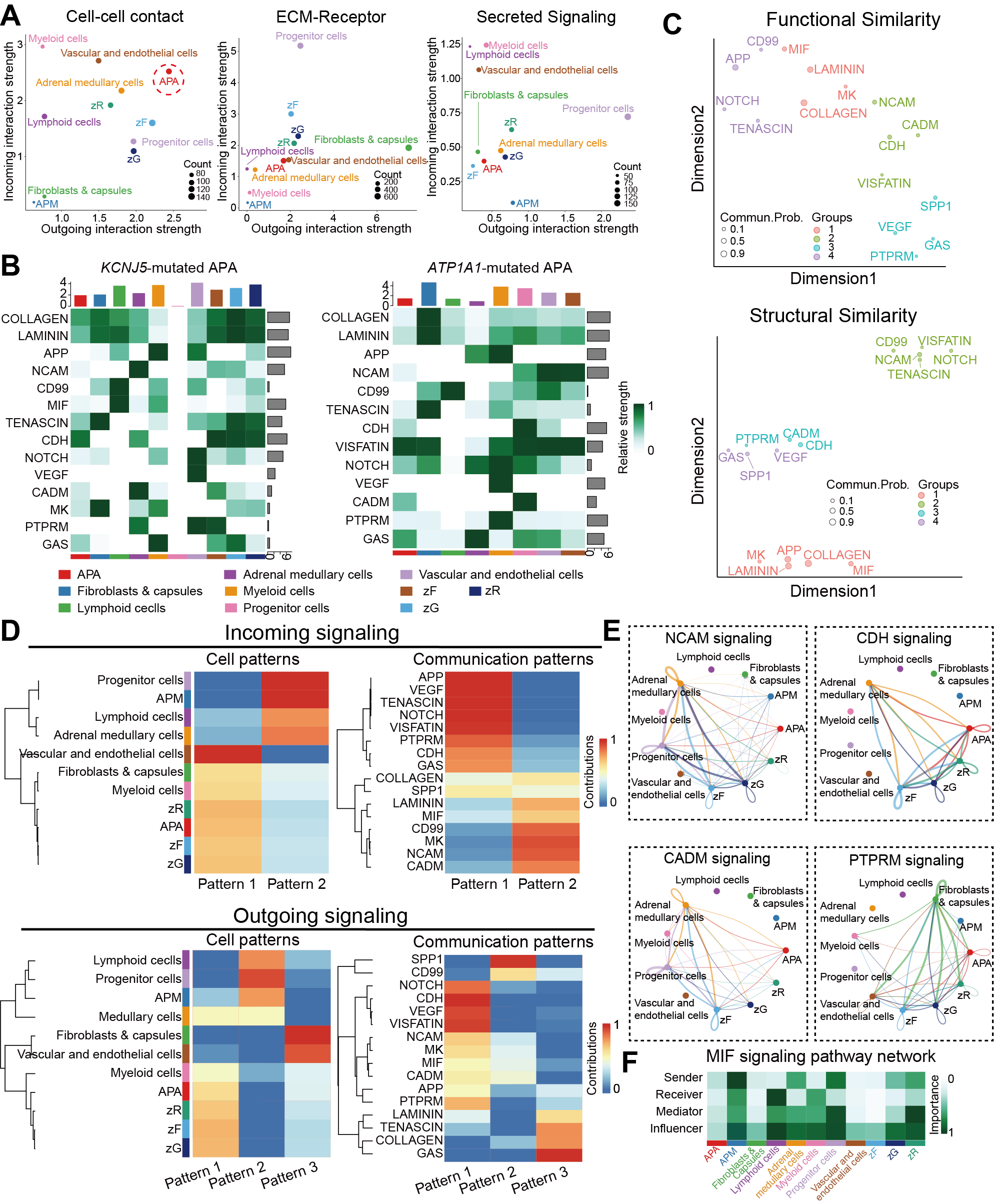
Figure S6. Cell-cell contact phenotype and preferential signaling pathways in APA cells**. Related to Figure 3 in the main manuscript: A) Cell-cell communication analysis identifies the number of incoming and outgoing signaling connections (left, cell-cell contact; middle, ECM-receptor; right, secreted) indicating APA cell-cell contact phenotype. B) Heatmap showing strength of outgoing and incoming interaction events between cell types across different APA genotypes (left, *KCNJ5*-mutated APA; right, *ATP1A1*-mutated APA). Top bars indicate total outgoing and incoming event intensities per cell type (labeled at bottom). Rows are ordered by signaling pathway; color intensity reflects interaction strength. C) Dimension reduction depicts functional and structural similarity of signaling pathways across cell types. D) Incoming (top) and outgoing (bottom) communication patterns, with contributions shown by cell type (left) and signaling pathway (right). E) Circle plot showing intercellular communication networks for four preferential signaling pathways. Thicker lines indicate more numerous and stronger interactions between cell types. F) Function of MIF (macrophage migration inhibitory factor) signaling pathway in cellular interaction. APA, aldosterone-producing adenoma; APM, aldosterone-producing micronodule; ECM, extracellular matrix; zF, zona fasciculata; zG, zona glomerulosa; zR, zona reticularis.


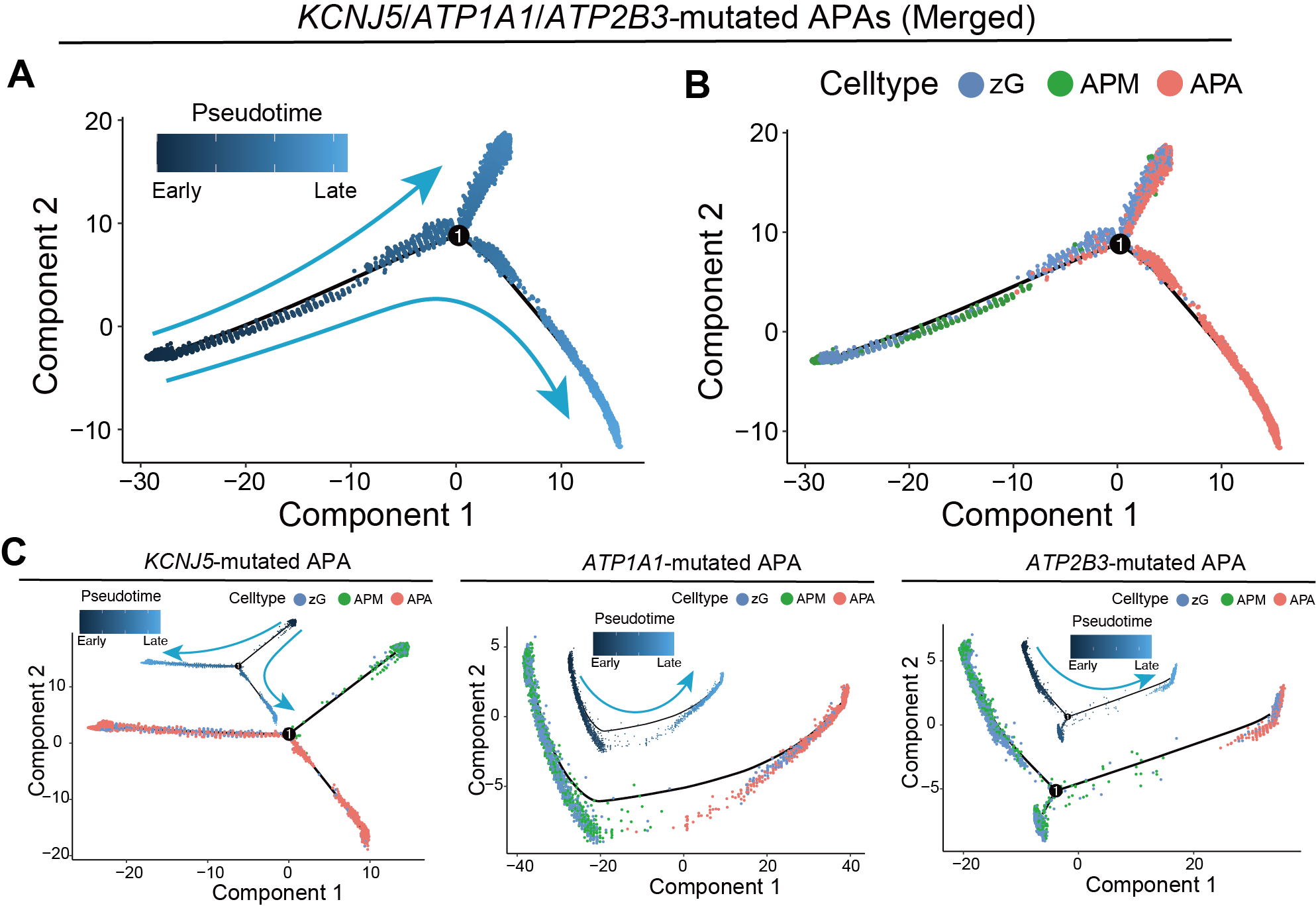


**Figure S7. Pseudotime trajectory analysis of zG, APM, and APA adrenal cell populations at the single-cell and single-nucleus level.** A,B) Reconstructed pseudotime trajectories of zG, APM, and APA cells inferred using Monocle2, based on two APM samples and 13 APA samples harboring *KCNJ5* or *ATP1A1* mutations, together with 5 peritumoral adrenal tissues adjacent to APA derived from cases with *KCNJ5* or *ATP2B3* mutations. The trajectory plot showing the direction of developmental time points arranged by pseudotime (A) and the three cell types assigned by different colors (B). Arrows indicating the likely course of evolution within the trajectory. C) Genotype-stratified pseudotime trajectories of *KCNJ5*-mutated APA (left), *ATP1A1*-mutated APA (middle), *ATP2B3*-mutated APA (right)). APA, aldosterone-producing adenoma; APM, aldosterone-producing micronodule; zG, zona glomerulosa. This figure relates to Figure 5 in the main manuscript.

**
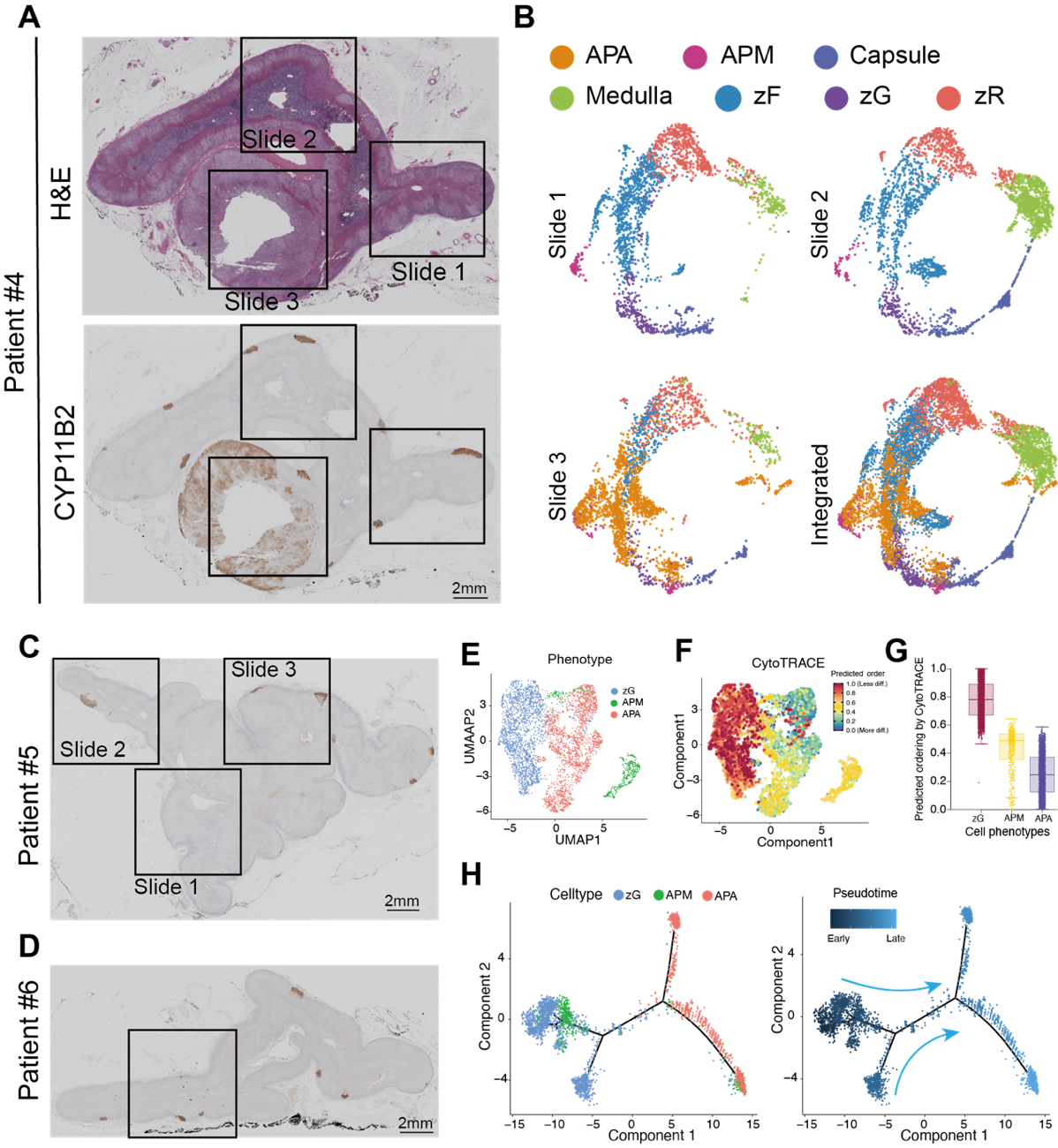
Figure S8. Pseudotime trajectory analysis of zG, APM, and APA cells at the spatial level in *KCNJ5*-mutated APAs.** Related to Figure 5 in the main manuscript: Captured regions from patient adrenal sections were used for spatial transcriptomics with the 10X Genomics Visium platform, and these regions were mapped onto adjacent sections stained with H&E or CYP11B2 IHC as indicated. A) three captured regions from patient #4 are shown, mapped onto H&E (top) or CYP11B2 IHC (bottom) stained sections. B) UMAP plot showing major spatial cell types in each captured and integrated region. C) three captured regions from patient #5, mapped onto a CYP11B2 IHC stained section. D) the single captured region from patient #6, mapped onto a CYP11B2 IHC stained section. E) UMAP plot of zG, APM, and APA cell distribution at the integrated transcriptome level. F) UMAP plot showing cell differentiation status using CytoTRACE, with a color scale from dark green (low stemness) to dark red (high stemness). G) box plot demonstrating predicted cell subpopulation ordering by CytoTRACE. H) reconstructed pseudotime evolution trajectory of zG, APM, and APA cells inferred by Monocle2, with the trajectory plot showing cell types by color (left) and developmental time points in pseudotime (right). Arrows indicate the likely course of evolution within the trajectory. APA, aldosterone-producing adenoma; APM, aldosterone-producing micronodule; H&E, hematoxylin and eosin; IHC, immunohistochemistry; zF, zona fasciculata; zG, zona glomerulosa; zR, zona reticularis.

**
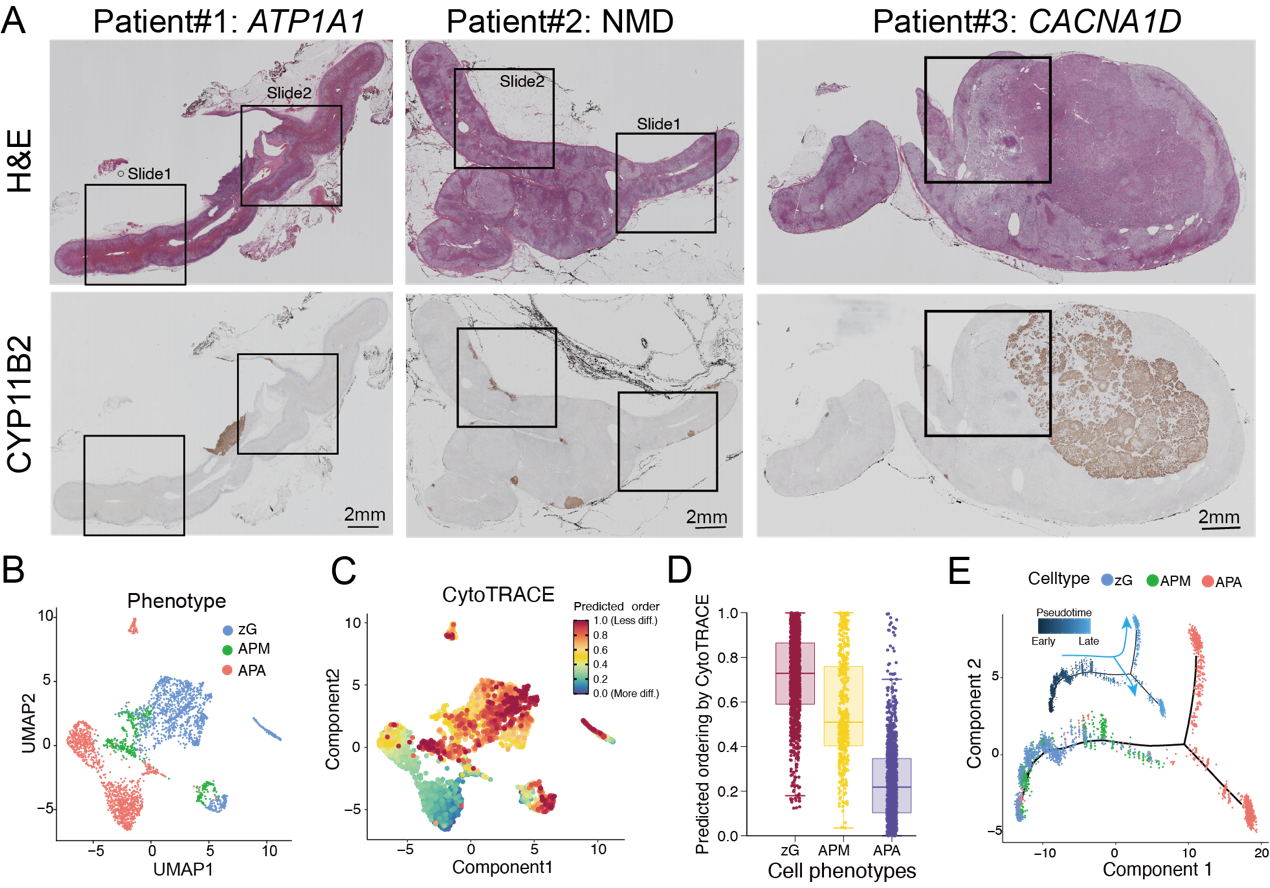
**

**Figure S9**. **Pseudotime trajectory analysis of zG, APM, and APA cells at the spatial level in APAs without a *KCNJ5* mutation.** Related to Figure 5 in the main manuscript: A) Spatial transcriptomic regions captured from three APA patients without *KCNJ5* mutations, including one *ATP1A1*-mutated, one *CACNA1D*-mutated, and one with no detectable mutations (NMD). Tissue sections were profiled using the 10X Genomics Visium platform, with corresponding H&E (top) and CYP11B2 immunohistochemistry staining (bottom). B) UMAP plot illustrating the distribution of zG, APM, and APA cells at the integrated transcriptome level. C) UMAP plot showing cell differentiation status using CytoTRACE. Color scale from dark green (low stemness) to dark red (high stemness) indicates differentiation levels. D) Box line plot demonstrated the predicted ordering by CytoTRACE of cell subpopulations. E) Reconstructed pseudotime evolution trajectory of zG, APM, and APA cells inferred by Monocle2. The trajectory plot showing the three cell types assigned by different colors and the direction of developmental time points arranged by pseudotime. APA, aldosterone-producing adenoma; APM, aldosterone-producing micronodule; H&E, hematoxylin and eosin; IHC, immunohistochemistry; NMD: no mutation detected in *KCNJ5*, *CACNA1D*, *ATP1A1 or ATP2B3*; zG, zona glomerulosa.

**
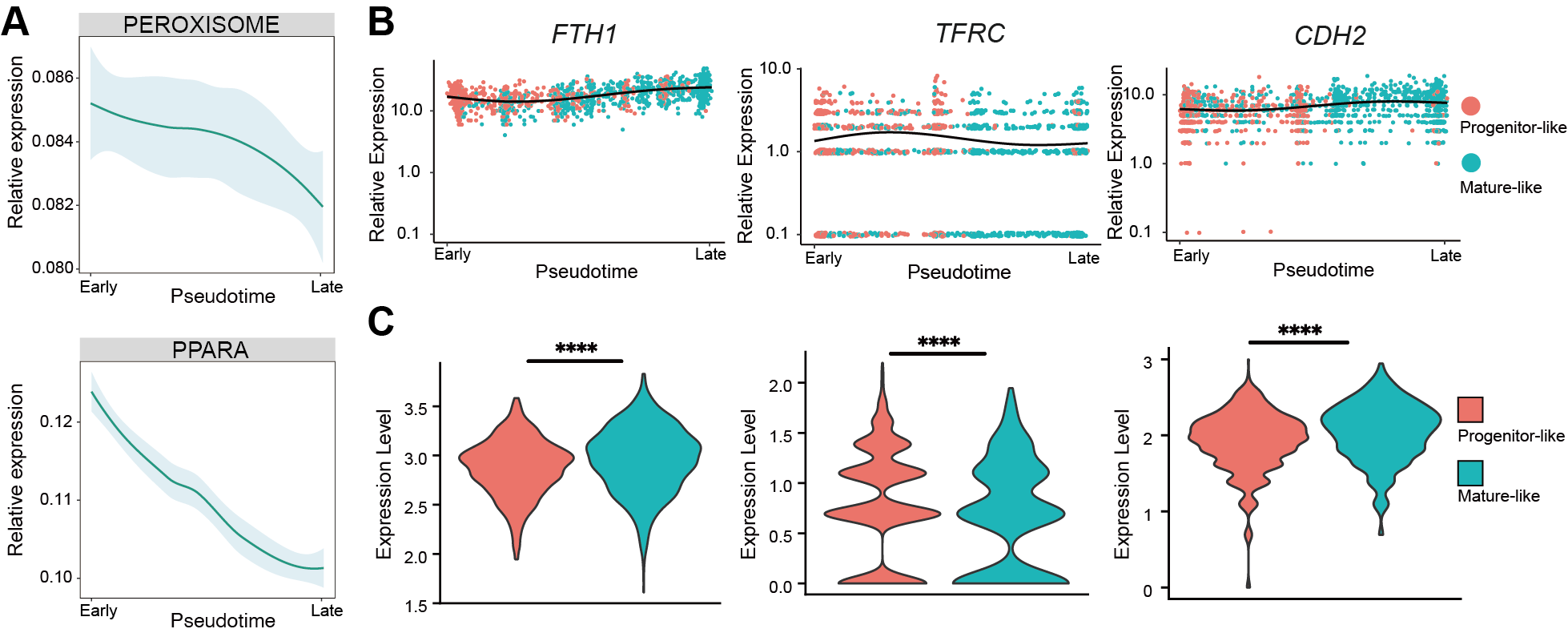
**

**Figure S10. Dynamic changes of biological processes and gene expression during APA progression.** Related to Figure 6 in the main manuscript: **A) Expression dynamics** of key biological processes during APA progression, including peroxisome (top) and peroxisome proliferator-activated receptor alpha (PPARA, bottom), showing changes over pseudotime. B) Pseudotime-ordered expression patterns of *FTH1* (left), *TFRC* (middle), and *CDH2* (right). C) Violin plot comparing the expression of *FTH1* (left), *TFRC* (middle), and *CDH2* (right) between progenitor-like and mature APA cell states. Statistical significance was determined using a two-sided Wilcoxon rank-sum test. *****P* < 0.0001.


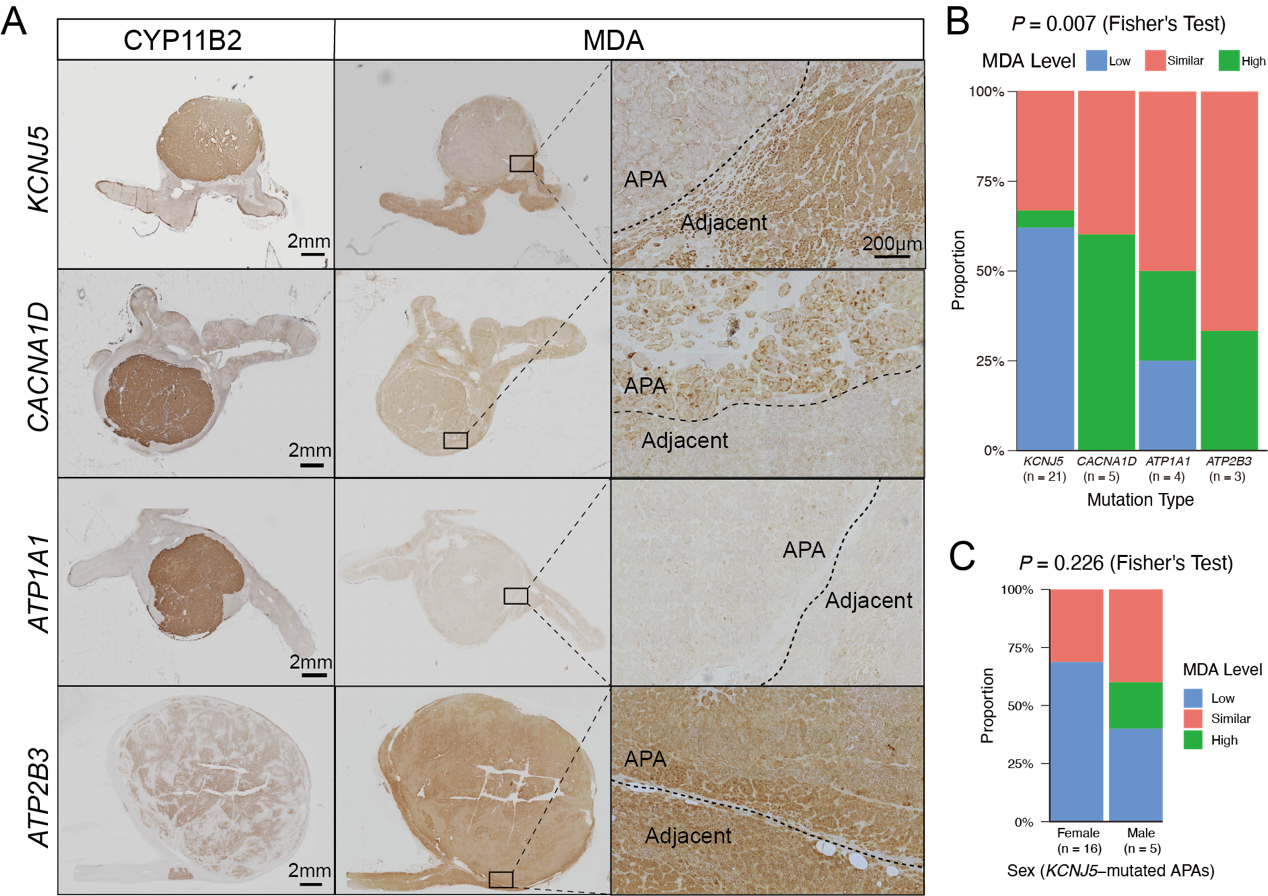


**Figure S11. Malondialdehyde immunohistochemistry of aldosterone-producing adenomas according to genotype.** Related to Figure 6 in the main manuscript: A) The Immunohistochemistry of CYP11B2 and MDA in adrenal sections with an APA-carrying mutation in either *KCNJ5* (n = 21), *CACNA1D* (n = 5), *ATP1A1* (n = 4) or *ATP2B3* (n = 3) as shown. A representative image for each APA-mutated gene is shown. Scale bars=500 µm. B), Quantification of MDA staining levels across APA samples with mutations in *KCNJ5*, *CACNA1D, ATP1A1,* or *ATP2B3*. *KCNJ5*-mutated APAs more frequently exhibited reduced MDA level. Statistical analysis was performed using Fisher’s exact test (*P* = 0.007). C) Comparison of MDA staining levels between male and female patients within the *KCNJ5*-mutated APA group. Statistical analysis was performed using Fisher’s exact test (*P* = 0.226). APA, aldosterone-producing adenoma; MDA, malondialdehyde.

**
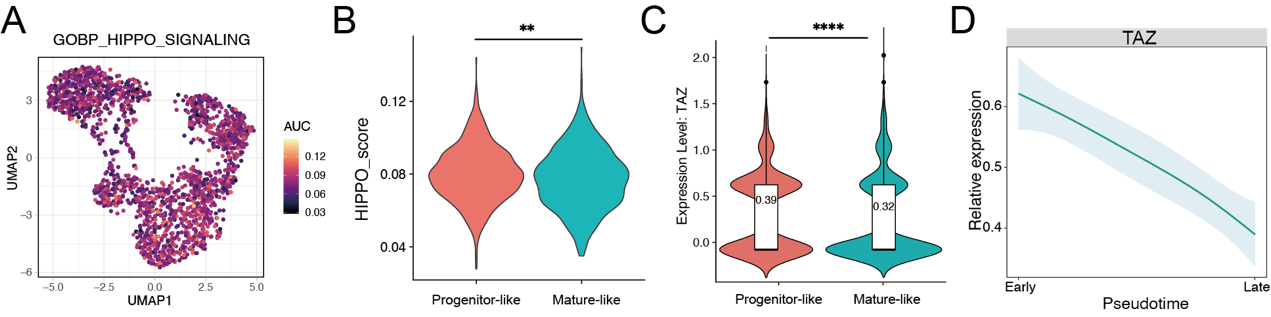
**

**Figure S12.** **Stage-specific Hippo signaling and *TAZ* expression dynamics along the APA pseudotime trajectory in spatial transcriptomic data.** Related to Figure 7 in the main manuscript: A) UMAP plot showing the distribution of Hippo signaling scores for each cell by AUCell. B) Violin plot comparing Hippo pathway scores between progenitor-like and mature APA cell states, with statistical significance determined by a two-sided Wilcoxon rank-sum test. C) Higher *TAZ* expression observed in progenitor-like regions, with statistical significance determined by a two-sided Wilcoxon rank-sum test. D) Decline in *TAZ* expression along the pseudotime trajectory, suggesting potential stage-specific regulation of Hippo signaling. ***P* < 0.01, *****P* < 0.0001. APA, aldosterone-producing adenoma.
